# Multicomponent Characterization of the Flower Bud of *Panax notoginseng* and Its Metabolites in Rat Plasma by Ultra-High Performance Liquid Chromatography/Ion Mobility Quadrupole Time-of-Flight Mass Spectrometry

**DOI:** 10.3390/molecules27249049

**Published:** 2022-12-19

**Authors:** Xiaonan Yang, Ying Xiong, Hongda Wang, Meiting Jiang, Xiaoyan Xu, Yueguang Mi, Jia Lou, Xiaohang Li, He Sun, Yuying Zhao, Xue Li, Wenzhi Yang

**Affiliations:** 1 State Key Laboratory of Component-Based Chinese Medicine, Tianjin University of Traditional Chinese Medicine, 10 Poyanghu Road, Tianjin 301617, China; 2 Haihe Laboratory of Modern Chinese Medicine, Tianjin University of Traditional Chinese Medicine, 10 Poyanghu Road, Tianjin 301617, China

**Keywords:** *Panax notoginseng*, flower bud, saponin, multicomponent identification, metabolism

## Abstract

The flower bud of *Panax notoginseng* (PNF) consumed as a tonic shows potential in the prevention and treatment of cardiovascular diseases. To identify the contained multi-components and, in particular, to clarify which components can be absorbed and what metabolites are transformed, unveiling the effective substances of PNF is of vital significance. A unique ultrahigh-performance liquid chromatography/ion mobility quadrupole time-of-flight mass spectrometry (UHPLC/IM-QTOF-MS) profiling approach and efficient data processing by the UNIFI^TM^ bioinformatics platform were employed to comprehensively identify the multi-components of PNF and the related metabolites in the plasma of rats after oral administration (at a dose of 3.6 g/kg). Two MS^2^ data acquisition modes operating in the negative electrospray ionization mode, involving high-definition MS^E^ (HDMS^E^) and data-dependent acquisition (DDA), were utilized aimed to extend the coverage and simultaneously ensure the quality of the MS^2^ spectra. As a result, 219 components from PNF were identified or tentatively characterized, and 40 thereof could be absorbed. Moreover, 11 metabolites were characterized from the rat plasma. The metabolic pathways mainly included the phase I (deglycosylation and oxidation). To the best of our knowledge, this is the first report that systematically studies the in vivo metabolites of PNF, which can assist in better understanding its tonifying effects and benefit its further development.

## 1. Introduction

*Panax notoginseng*, a genuine medicinal material mainly distributed in Yunnan, China, is considered to be “more valuable than gold” in the Materia Medica, which is also the main raw material for some well-known Chinese patent medicines (CPMs), such as “Yunnan Baiyao”, “Pianzaihuang”, “Xueshuantong Injection”, “Qiye Shen’an Tablet”, “Compound Danshen Dropping Pill”, etc. [1]. Specifically, *P. notoginseng* has been proven to have the functions of hemostasis, blood lipid reduction, antithrombosis, immunological enhancement, anticancer, etc. [2,3]. Due to the remarkable tonic effect, *P. notoginseng* is also widely used for the purpose of health care [4]. Among the multiple classes of components ever reported from *P. notoginseng*, the saponins are considered as one of the most important compounds closely related to the pharmacological effects [5]. *Panax notoginseng* saponins could inhibit the growth of a variety of tumor cells and regulate the oxidative stress of immune cells induced by PCV2 infection in vitro and in vivo [6,7]. Aside from the rhizome and root as the official source for the medicinal use, the other parts of *P. notoginseng*, such as the leaf (8.53% ± 0.92% saponins [8]) and the flower bud (> 20.00% saponins [9]) contain saponins, as well. In particular, the flower bud of *P. notoginseng* (PNF) is rich in protopanaxadiol ginsenosides [10]. PNF has anti-inflammatory, analgesic, sedative, hypolipidemic, antihypertensive, and other activities [11]. Additionally, PNF can be consumed as tea domestically and exported overseas with its excellent taste [8]. Most of the research on PNF focuses on its chemical composition and pharmacological effects, and the quality standard of PNF is only included in local standards in Shanghai, Chongqing, Sichuan, etc. However, to the best of our knowledge, no report is currently available on its in vivo metabolism. To clarify what in PNF can be absorbed and what its metabolites can be transformed into is of great significance to unveiling its main bioactive components, which thus benefits the development of more healthcare products and its holistic quality control.

Due to the diversity and complexity of the chemical components for traditional Chinese medicine (TCM), comprehensive multicomponent characterization is still encountered with great challenge [12]. In recent years, great advances have been made because of the rapid development of instrumentation and in silico data processing vehicles [13]. Liquid chromatography mass spectrometry (LC-MS), especially ultrahigh-performance liquid chromatography/high-resolution mass spectrometry (UHPLC/HRMS), has become the most widely used tool in support of herbal component analysis and quality evaluation [14,15,16,17]. Versatile multidimensional liquid chromatography (MDC) can increase the peak capacity and improve the selectivity of separation, which thereby enables in-depth multicomponent characterization (such as comprehensive two-dimensional liquid chromatography) and targeted quantitative assays of TCM markers of interest (multi-heart cutting two-dimensional liquid chromatography) [18,19]. In addition, hybrid high-resolution MS contributes to an accurate MS^n^ (*n* ≥ 2) measurement, different scanning methods, and alternative fragmentation modes [20,21]. Typically, multicomponent characterization for TCM is performed by data-dependent acquisition (DDA) or data-independent acquisition (DIA) in a non-target mode [22,23,24]. In contrast, DDA requires no prior knowledge capable of the automated MS^n^ acquisition based on some criteria (such as the intensity ranking top N setting) by collision-induced dissociation (CID) or high-energy collision-induced dissociation (HCD) [25,26,27,28]. The MS^n^ spectra obtained by DDA are of high quality and easier to interpret, rendering DDA more preferably utilized. However, due to the complex chemical composition of TCM, some factors, such as the adducts, dimers, in-source fragments of the precursor ions, and diverse interference ions can lead to the repeated and noneffective MS^n^ data recording, and thus damage the coverage on the interested components. Otherwise, the DIA mode can obtain the secondary fragmentation information of all ions in the predefined mass window [29], but the matching between the precursors and product ions is inevitable, which can be achieved using commercial software (such as UNIFI^TM^ from Waters) or inhouse-developed algorithms [30,31]. Furthermore, the introduction of ion mobility spectroscopy (IMS) allows for the additional dimension of ion separation based on the charge state, size, and shape [32]. Hybrid ion mobility/quadrupole time-of-flight mass spectrometry (IM/QTOF-MS) coupled with liquid chromatography (LC) allows up to three-dimensional separation, providing four-dimensional information related to the structure of the analytes (including *t*_R_, drift time or collision cross section-CCS, MS^1^ and MS^2^), which can demonstrate a potent vehicle for herbal component analysis [27,33].

**Figure 1:**
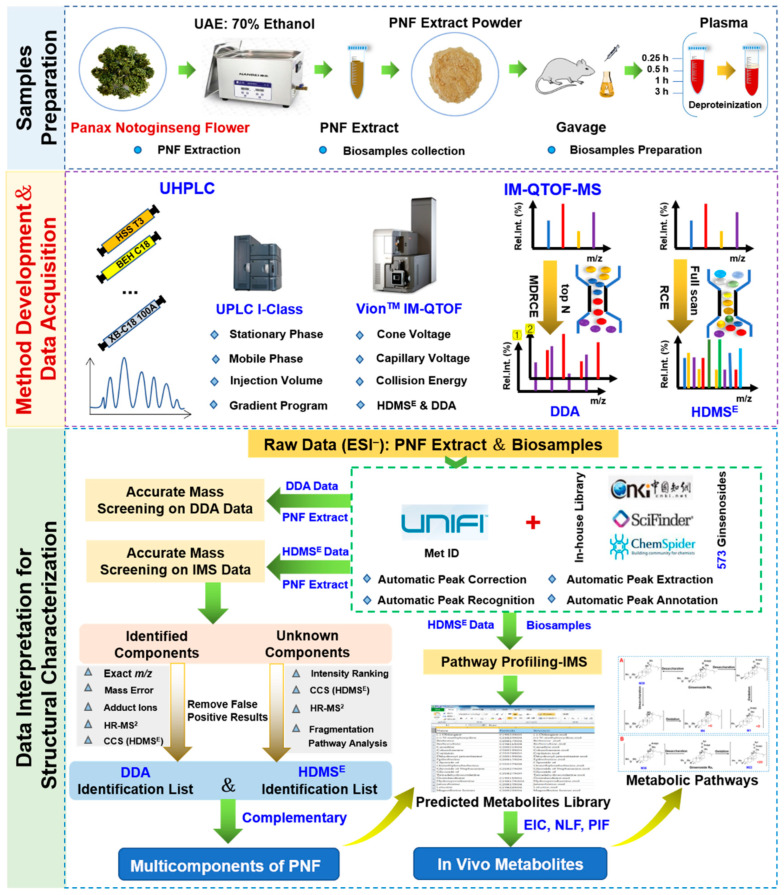
General technical flowchart for the comprehensive characterization of the multi-components from *Panax notoginseng* flower bud and its in vivo metabolites in rat plasma by UHPLC/IM-QTOF-MS and intelligent data processing facilitated by UNIFI^TM^.

**Figure 2:**
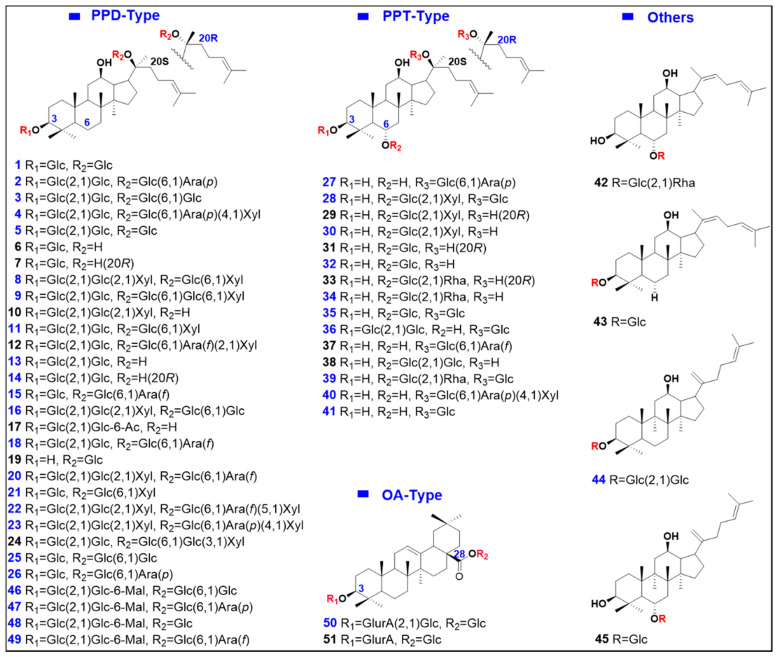
Chemical structures of 51 reference compounds of ginsenosides (the components identified from the PNF extract by comparison with reference compounds are marked with the numbers in bule).

The aim of this work was to identify the multi-components of PNF and those that could be absorbed by rats by developing the UHPLC/IM-QTOF-MS approaches. In particular, to balance between the coverage on PNF components and the MS spectral quality, by utilizing the Vion^TM^ IM-QTOF LC-MS platform, we integrated two data acquisition modes, DDA and HDMS^E^ (an IM separation enabled DIA approach) for the untargeted MS^2^ spectra acquisition in the negative mode [34,35]. Moreover, intelligent data processing was enabled with the workflows of the UNIFI^TM^ bioinformatics platform, and the searching of an in-house ginsenoside library (recording 573 known ginsenosides) assisted to rapidly annotate the ginsenoside components and their metabolites from the PNF extract and rat plasma. The overall technical roadmap was shown in Figure 1 and the chemical structures of 51 reference compounds were shown in Figure 2 (with the information detailed in Table S1. Notably, to gain the satisfactory performance, we had carefully optimized key factors/parameters affecting the reversed-phase UHPLC separation and MS detection on Vion^TM^ IM-QTOF, in the current work. The established analytical approach accomplished both the multicomponent characterization of PNF and the identification of prototype compounds/metabolites in rat plasma, demonstrating high applicability. Additionally, the discrimination of isomeric metabolites by CCS prediction was discussed.

## 2. Results and Discussion

### 2.1. Development of a UHPLC/IM-QTOF-MS Approach to Separate and Characterize the Multi-Components from PNF by Integrating the Acquisition of HDMS^E^ and DDA Data

Due to the complexity and variability of the components of TCM, the analytical strategy that can enable the identification of the multiple classes of components is particularly important [12,36]. To achieve good separation and the comprehensive characterization of the saponins in PNF, the Vion^TM^ IM-QTOF hybrid high-resolution mass spectrometry in combination with UHPLC was used in the current work. In particular, two MS^2^ data acquisition methods, involving the IM separation-enabled data-independent HDMS^E^ and data dependent acquisition (DDA), were developed aimed to record more fragmentation information of the PNF components useful for the structural elucidation [34]. On the basis of our previous work, the total extract of PNF was utilized to optimize the UHPLC condition and key MS parameters set for HDMS^E^ and DDA.

**Figure 3:**
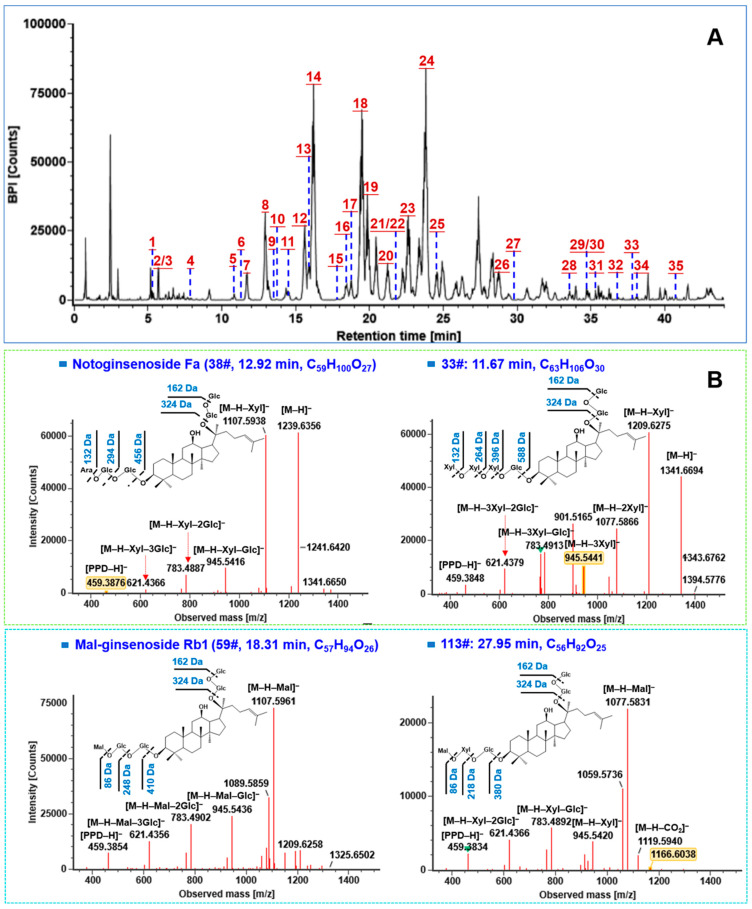
Structural elucidation of ginsenosides from PNF. (**A**) The based peak chromatogram of PNF obtained in the negative ESI mode by UHPLC/IM-QTOF-MS (those identified by comparison with the reference standards are annotated: **1**, noto-R1; **2**/**3**, Rg1/Re; **4**, vina-R4; **5**, noto-R4; **6**, noto-R2; **7**, chiku-L5; **8**, noto-Fa; **9**, Rg2; **10**, Rh1; **11**, F3; **12**, noto-FP2; **13**, noto-Rh1; **14**, Rb1; **15**, Ro; **16**, m-Rb1; **17**, noto-Fc; **18**, Rc; **19**, Ra1; **20**, noto-S; **21**/**22**, F1/m-Rc; **23**, Rb2; **24**, Rb3; **25**, m-Rb2; **26**, Rd; **27**, m-Rd; **28**, gypenoside XVII; **29**/**30**, noto-Fe/noto-Fd; **31**, Rd2; **32**, F2; **33**, Rg3; **34**, 20(*R*)-Rg3; **35**, Rk1); (**B**) analysis of the CID-MS^2^ spectra for the representative ginsenosides of the PPD type and malonylated type.

For UHPLC separation in the reversed-phase mode, our previous studies regarding ginsenoside analysis could demonstrate the addition of formic acid (FA, 0.1%) in both the organic and water phases of the mobile phase benefitted the separation and monitoring of ginsenosides by MS in the negative ESI mode [25]. In addition, the stationary phase is an important factor affecting the resolution of PNF components in the reversed-phase mode. In this work, 20 commercial chromatographic columns, including HSS T3, HSS C18 SB, CSH C18, BEH Shield RP18, BEH C18, Atlantis Premier BEH C18 AX, Cortecs C18, CSH Phenyl-Hexyl, CORTECS UPLC C18+, CORTECS UPLC Shield RP18, CSH Cyano, CSH Fluoro-Phenyl (from Waters, Milford, MA, USA), Zorbax Extend C18, Zorbax SB C18, Zorbax Eclipse Plus C18, Zorbax SB Aq (from Agilent, Santa Clara, CA, USA), and Luna Omega Polar C18, Kinetex Biphenvl, Kinetex XB-C18 (from Phenomenex, Torrence, CA, USA), and Exsil Pure C18 (from Exmere Ltd., Carnforth, UK), were evaluated. In this regard, the number of resolvable peaks, the overall resolution and distribution of chromatographic peaks, were taken into account as the index for the assessment. The results showed that the HSS T3, Zorbax Eclipse Plus C18, BEH C18, and CSH C18 columns could resolve more peaks than the other columns under the same UHPLC/IM-QTOF-MS condition (Figure S1 from Supplementary Materials ). And amongst them, the overall distribution of chromatographic peaks was good on the Zorbax Eclipse Plus C18 column, while the peak on the HSS T3 column displayed better symmetry. Moreover, ginsenosides with similar polarity could be evenly distributed (PPT-type and PPD-type were eluted first, and the acidic saponins were eluted later). We therefore selected the HSS T3 column for the subsequent experiments, consistent with one of our previous works [37]. On this basis, by slightly adjusting the gradient elution procedure, satisfactory separation of PNF was achieved within 45 min. Figure 3A showed the base peak chromatogram (BPC) of the PNF extract in the negative ESI mode.

In the next step, we optimized the key ion source parameters of the Vion^TM^ IM-QTOF instrument (Waters Corporation, Milford, MA, USA) in negative ESI mode. According to our previous study, capillary voltage, which mainly affected the ion response, was set at 1.0 kV, and cone voltage, advancing the ions and having the possibility to induce in-source fragmentation, was set at 20 V, respectively [24]. Collision energy for both HDMS^E^ and DDA had been previously optimized targeting the ginsenosides, and ramp collision energy (RCE) of 40–80 eV for HDMS^E^, and mass-dependent ramp collision energy (MDRCE) of 10–35/65–90 eV for DDA, were set. In DDA, the setting of top N criteria could largely influence the acquisition of useful MS^2^ spectra, and thus determined the number of components that could be characterized [27,28]. Here, we evaluated three different top N settings (top1, top2, and top3) by comparing the components primarily characterized based on the UNIFI results (automated peak annotation by searching the inhouse ginsenoside library to generate the tables of “Identified Components” and “Unknown Components”). In contrast, by the top3 criteria, the largest number of primarily characterized components was enabled (Figure S2 from Supplementary Materials), which could enable the characterization of more components from PNF, and thereby was selected in DDA.

### 2.2. In-Depth Characterization of Ginsenosides from PNF by Analyzing the Negative DDA and HDMS^E^ via the Intelligent UNIFI Workflows

The bioinformatics platform, UNIFI, was used to process the obtained high-accuracy DDA and HDMS^E^ data, achieving the efficient, intelligent, and comprehensive characterization of PNF components. Automated data correction and peak annotation were enabled by applying the “Screening” analysis and searching the inhouse library consisting of 573 ginsenosides. Two tables including the “Identified” and “Unknown” components were obtained. On the one hand, the “Identified Components” section contained the identifications that were consistent with the inhouse ginsenoside library, conforming to the predefined matching tolerance. It displayed multiple information regarding each characterized component (e.g., the identification, observed *m/z*, formula, observed *t*_R_, mass error, and adduct, etc. On the other hand, the “Unknown Components” section was composed by a list of the extracted components that failed to match with the inhouse library [24,26,30,32,33,37,38]. Both the DDA and HDMS^E^ data were analyzed to expand the components that could be characterized from PNF, by adopting the following analysis strategy. Firstly, we analyzed the DDA data, as it provided more reliable MS^2^ data with mass unit-resolved selection of the precursors. For each listed as the “Identified Components”, confirming analysis was performed by analyzing the adduct ions, excluding those false positives (caused by the adduct ions or in-source fragment ions), and MS/MS data interpretation to analyze the fragmentation pathways. For the “Unknown Components” part, the components in the table were sorted by the response size, and some false positives with the same retention time, response < 10,000, and invalid data were removed. In addition, the remaining components were manually identified based on the high-accuracy MS^1^ and MS^2^ data to enrich the identification results. The same procedures were followed to analyze the HDMS^E^ data. The final identification list was generated according to these operations: (1) the CCS information deduced from the HDMS^E^ data was added to the compounds identified by DDA; (2) the components newly identified by HDMS^E^ were added. Integration of the analysis of both the DDA and HDMS^E^ data exerted remarkable complementarity beneficial to the characterization of more ginsenoside components from PNF. As a result, a total of 219 ginsenosides got identified or tentatively characterized from PNF, and by searching the inhouse library, 121 compounds thereof (accounting for 59.82% of the total amount) may have not been isolated from the entire *Panax* genus. Table S2 from Supplementary Materials detailed the information for these 219 components, containing 35 ones unambiguously identified by comparison with the reference standards. When being classified according to the sapogenin and typical substituent, they could be divided into 83 PPD-type, 26 PPT-type, 105 malonylginsenosides, and 5 others.

#### 2.2.1. Characterization of PPD-/PPT-Type Ginsenosides

In the current work, a total of 83 PPD-type ginsenosides (37.7%) and 26 PPT-type ginsenosides (26.90%) were characterized from the PNF extract, which were the neutral saponins (carboxyl-free) representing the most common ginsenoside subtypes for the *Panax* species [24,39]. The precursor ions for PPD- and PPT-type ginsenosides were featured by the predominant FA-adduct ([M–H + HCOOH]^−^), and their negative CID-MS^2^ showed the continuous neutral loss of the sugar moiety and the formation of typical deprotonated sapogenin or related secondary product ions (cleavage of C_6_H_12_ from the C-17 side chain) [40,41]. The characteristic neutral loss corresponding to the glycosyl moiety included glucuronic acid (GlurA, 176.03 Da), glucose (Glc, 162.05 Da), rhamnose (Rha, 146.06 Da), and arabinose/xylose (Ara/Xyl, 132.04 Da) [23,24,41]. The diagnostic product ions (DPIs) enabling the rapid identification of the PPD-type and PPT-type saponins were *m/z* 459.38 ([PPD−H]^−^)/375.29 ([PPD–H–C_6_H_12_]^−^) and *m/z* 475.38 ([PPT–H]^−^)/391.29 ([PPT–H–C_6_H_12_]^−^), respectively [41]. These fragmentation features could be reflected by a PPD-type ginsenoside, notoginsenoside Fa (corresponding to **38#**: *t*_R_, 12.92 min; C_59_H_100_O_27_) with five sugars glycosylated at the 3- and 20-OH of the sapogenin. Due to the use of MDRCE of 10–35/65–90 eV, a rich series of product ions at *m/z* 1239.6357 ([M–H]^−^), 1107.5938 ([M–H–Xyl]^−^), 945.5416 ([M–H–Xyl–Glc]^−^), 783.4887 ([M–H–Xyl–2Glc]^−^), 621.4366 ([M–H–Xyl–3Glc]^−^), and 459.3876 ([M–H–Xyl–4Glc]^−^/[PPD–H]^−^), were dissociated from the FA-adduct precursor ion of *m/z* 1285.6412, which indicated the cleavage of four Glc and one Ara moieties from the genuine deprotonated precursor of *m/z* 1239, respectively (Figure 3B). For the unknown compound **33#** (*t*_R_, 11.67 min; C_63_H_106_O_30_), the characteristic product ion at *m/z* 459.3848 was observed in the MS^2^ spectra, which could indicate a PPD-type ginsenoside. The other product ions dissociated from the precursor at *m/z* 1387.6756, involving *m/z* 1341.6694 ([M–H]^−^), 1209.6275 ([M–H–Xyl]^−^), 1077.5866 ([M–H–2Xyl]^−^), 945.5441 ([M–H–3Xyl]^−^), 783.4914 ([M–H–3Xyl–Glc]^−^), 621.4379 ([M–H–3Xyl–2Glc]^−^), and 459.3848 ([M–H–3Xyl–3Glc]^−^/[PPD–H]^−^), were observed, which indicated the attachment of three Glc and three Xyl residues (we used Xyl to represent all the pentose moiety characterized by the neutral loss of 132.04 Da for the convenient description of the structures) on the PPD sapogenin. According to the above information, we preliminarily characterized compound **33#** as notoginsenoside Q or Fh1 or isomer (PPD-3Xyl-3Glc), as shown in Figure 3B.

#### 2.2.2. Characterization of Malonyl Ginsenosides

The PPD- and PPT-type ginsenosides in the *Panax* genus are prone to diverse acylation reactions to generate the malonyl (C_3_H_2_O_3_; 86.00 Da), dimalonyl (C_6_H_4_O_6_; 172.09 Da), acetyl (C_2_H_2_O; 42.01 Da), and crotonyl (C_4_H_4_O; 68.03 Da) derivatives [39]. Among them, malonyl ginsenosides are a class of rich acidic saponins especially in the flower part [42,43,44]. Different from the neutral PPD-/PPT- subtypes, the full-scan MS^1^ spectrum for malonyl ginsenosides showed the companied precursor of [M–H]^−^ and the [M–H–CO_2_]^−^ in-source decay fragment. Moreover, the negative CID-MS^2^ of malonyl ginsenosides could easily eliminate the entire malonyl group by neutral loss of C_3_H_2_O_3_ (−86.00 Da) and C_3_H_2_O_3_ + H_2_O (−104.01 Da) together with a series of product ions similar to the neutral glycosyl loss occurring for the PPD-/PPT-type ginsenosides [42,44]. We could illustrate these fragmentation features by analyzing the CID-MS/MS fragmentation of the reference compound, m-Rb1 (consistent with component **59#**, *t*_R_, 18.31 min; C_57_H_94_O_26_). It provided the deprotonated precursor ion at *m/z* 1193.5966 ([M–H]^−^), which, upon CID-MS/MS fragmentation, yielded the fragment ions at *m/z* 1107.5965 ([M–H–Mal]^−^) in the high-mass region of the MS^2^ spectrum, with neutral loss of the malonyl group. The other fragment ions, at *m/z* 945.5437 ([M–H–Mal–Glc]^−^), 783.4904 ([M–H–Mal–2Glc]^−^), 621.4378 ([M–H–Mal–3Glc]^−^), and 459.3853 ([M–H–Mal–4Glc]^−^/[PPD–H]^−^), were similar to those dissociated from ginsenoside Rb1 (Figure 3B). These fragmentation features assisted to characterize an unknown compound **113#** (*t*_R_, 27.95 min; C_56_H_92_O_25_), which provided the [M–H]^−^ ion at *m/z* 1163.5853. The fragment at *m/z* 1119.5940 should result from the deprotonated precursor by losing CO_2_ ([M–H–CO_2_]^−^), while the neutral saponin structure was consistent with the fragment of *m/z* 1077.5831 ([M–H–Mal]^−^). The other fragments, including *m/z* 945.5420 ([M–H–Mal–Xyl]^−^), 783.4892 ([M–H–Mal–Xyl–Glc]^−^), 621.4264 ([M–H–Mal–Xyl–2Glc]^−^), and 459.3834 ([M–H–Mal–Xyl–3Glc]^−^/[PPD–H]^−^), could inform the presence of three Glc and one Xyl on the PPD sapogenin, similar to the common ginsenosides Rc, -Rb2, and -Rb3. This evidence could render the tentative characterization of compound **113#** as PPD-3Glc-Xyl-Mal. Because of the difference of retention time with m-Rc (74#: *t*_R_, 21.68 min) and m-Rb2 (96#: *t*_R_, 24.67 min), compound **113#** matched with malonyl floral ginsenosides Rc1/-Rc2/-Rc3/-Rc4 in the inhouse library. However, we were in need of more dimension of information to discriminate among these ginsenoside isomers.

**Figure 4:**
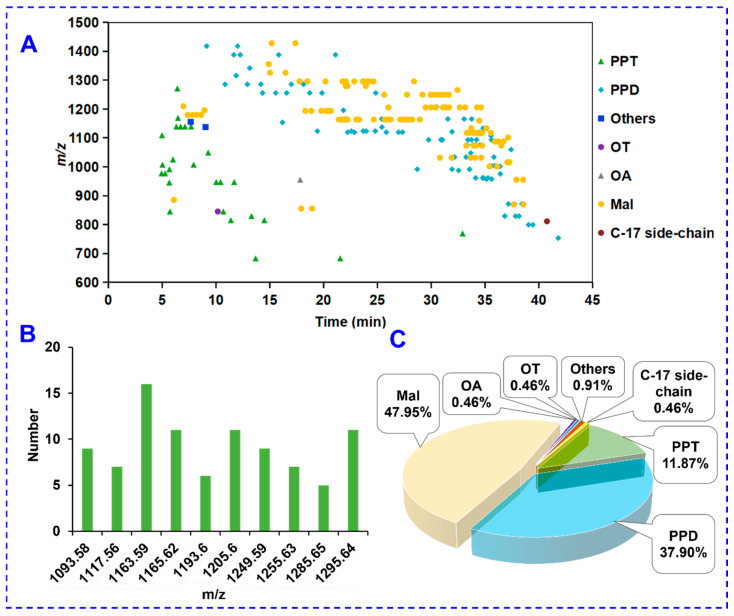
Summary on the ginsenosides characterized from PNF: (**A**) a two-dimensional scatter plot by *t*_R_ and *m/z* showing the relationship between the retention time and molecular mass; (**B**): a bar chart showing the numbers of typical ginsenoside isomers; (**C**): a pie chart sorted by the ginsenoside subtypes.

The structural features for the characterized PNF saponins were analyzed. Figure 4A showed a 2D scatter plot of 219 saponins with *t*_R_ (min) and *m/z* as the horizontal and vertical coordinates. As a feature, the saponins in PNF had large molecular weight. In detail, 165 saponins exhibited *m/z* > 1000 (75.3% of the total amount), 120 ones with *m/z* > 1100 (54.8%), 61 with *m/z* > 1200 (27.9%), and 12 with *m/z* > 1300 (5.5%). In addition, isomerism was very common for the PNF saponins, and Figure 4B showed the numbers of the top ten most isomers. Notably, the saponins with *m/z* 1163.59 reached up to 16. In addition, the saponins characterized from PNF mainly involved the malonylated, PPT-and PPD-types. In particular, the PPD-type and malonyl ginsenosides were abundant in PNF. Figure 4C confirmed this feature by the proportion of different ginsenoside subtypes, and evidently, ginsenosides of the other common subtypes (e.g., the OA-type, OT-type, and C-17 side chain-varied were very few. These results, in general, were consistent with previous reports [25,37,42].

### 2.3. Identification of the Absorbed Components in Rat Plasma

Identification of the components of PNF that could be absorbed and the transformed metabolites are crucial to better understanding the effective components of PNF. On the basis of the multicomponent characterization results as aforementioned, the same UHPLC/IM-QTOF-MS method was used to collect the data of rat plasma after administrating the extract solution of PNF. By comparing the BPI chromatograms of the three dose groups, the high-dose group (at the dose of 3.6 g/kg) was selected, having most chromatographic peak information. The UNIFI Met-ID was employed to identify the metabolites in vivo. Here, a total of 51 components were absorbed in the rat plasma, including 40 prototype compounds (Table S3 from Supplementary Materials) and 11 metabolites (Table S4 from Supplementary Materials).

#### 2.3.1. Prototype Component Identification

**Figure 5:**
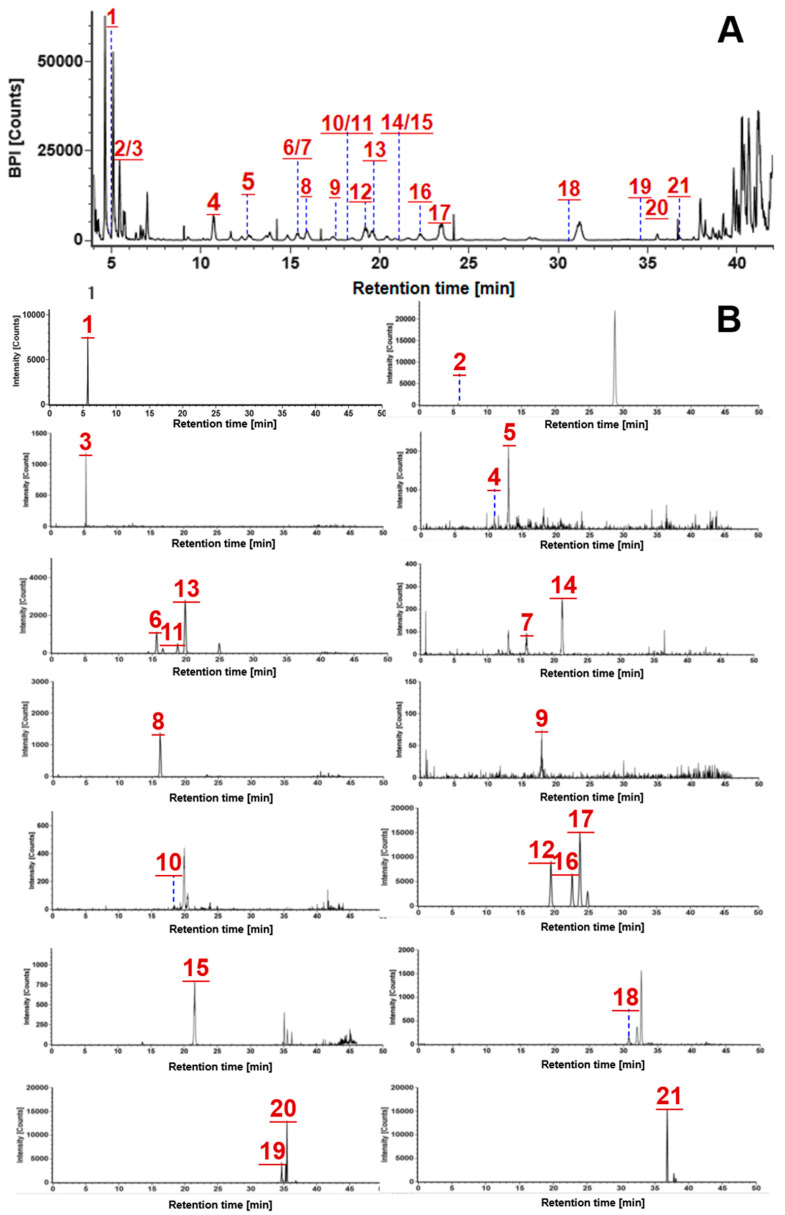
Characterization of the prototype components in rat plasma: (**A**)—the base peak chromatogram of PNF-administrated rat plasma; (**B**)—the extracted ion chromatogram (EIC) for those identified with the aid of reference compounds (**1**, noto-R1; **2**, Re; **3**, Rg1; **4**, noto-R4; **5**, noto-Fa; **6**, noto-FP2; **7**, noto-Fh1; **8**, Rb1; **9**, Ro; **10**, m-Rb1; **11**, noto-Fc; **12**, Rc; **13**, Ra1; **14**, noto-S; **15**, F1; **16**, Rb2; **17**, Rb3; **18**, m-Rd; **19**, noto-Fe; **20**, Rd_2_; **21**, F2).

Identification of the prototype compounds from rat plasma was performed by comparing with the data of PNF components recorded under the same conditions. Those common in the base peak chromatograms of PNF-administrated rat plasma and PNF extract, but absent in the blank sample, were characterized as the prototype compounds. Taking the determination of the prototype component **50#**-ginsenoside Rb1 (*t*_R_, 16.10 min; C_54_H_92_O_23_) as an example, the molecular ion peak of the compound appeared at *t*_R_ 16.10 min in the BPC of the dosed group, but not in the blank sample. It delivered the [M–H + HCOOH]^−^ precursor ion at *m/z* 1153.6020 in the MS^1^ spectrum, and characteristic fragments at *m/z* 1107.5950, 1089.5862, 945.5424, 783.4899, 621.4378, and 459.3842 in the MS^2^ spectrum. These data could demonstrate the identification of Rb1 as a prototype component in rat plasma from PNF. Among the 40 prototype compounds (Table S3 from Supplementary Materials), 21 thereof were identified because of the availability of reference compounds comparison. Figure 5A showed the prototype components in the BPC chromatogram of rat plasma as determined by the reference compounds, and each was exhibited by the extracted ion chromatogram (EIC) in part B of Figure 5. The MS/MS spectra of 18 prototype components were shown in Figure S3 from Supplementary Materials (noto-R4, Ro, and m-Rb1 could not display the MS/MS spectra due to low response).

#### 2.3.2. Identification of the In Vivo Metabolites

The in vivo metabolites in rat plasma were characterized based on the data of the high-dose group (3.6 g/kg). Predicted information for the potential metabolites was established by the Met-ID function of UNIFI based on 21 prototype components that were identified by comparison with the reference standards, while mass defect filter (MDF), neutral loss filter (NLF), and common fragment search (CFS), were used to screen the detectable metabolites from the complex biological matrix [43].

As a result, a total of 11 components from the rat plasma were identified as metabolites, with their information detailed in Table S4 from Supplementary Materials. Here, the deglycosylated metabolite **M4** was taken as an example for illustration. At 19.35 min in the MS^1^ spectrum, the molecular ion peak of **M4** (C_53_H_90_O_22_) appeared *m/z* 1077.5820 ([M–H]^−^), which was predicted by the UNIFI pathway to be the metabolite of notoginsenoside Fc (*t*_R_, 18.40min) by loss of xylose or of notoginsenoside Fa (*t*_R_, 12.84min) by loss of glucose. Its CID-MS^2^ product ions, such as *m/z* 945.5422 ([M–H–Xyl]^−^), 783.4883 ([M–H–Xyl–Glc]^−^), 621.4358 ([M–H–Xyl–2Glc]^−^), and 459.3844 ([M–H–Xyl–3Glc]^−^/[PPD–H]^−^) in the MS^2^ spectrum suggested the attachment of three Glc and one pentose on the PPD sapogenin. By searching the inhouse library of ginsenosides, **M4** was speculated to be vinaginsenoside R7. Compared with the prototype components, notoginsenosides Fc and -Fa, the retention time of **M4** was prolonged, which could result from the decrease of polarity due to deglycosylation.

**Figure 6:**
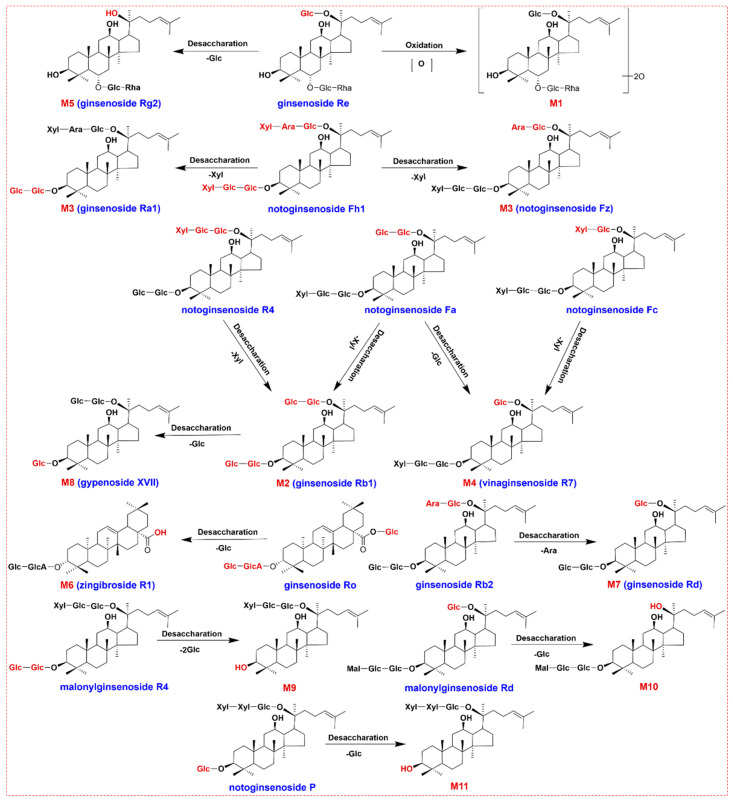
Proposed metabolic pathways for the ginsenosides of PNF.

The metabolic pathways of PNF in rat plasma were deduced, which was characterized by the phase I metabolic reactions of deglycosylation (**M2**–**M11**) and oxidation (**M1**), as shown in Figure 6. Amongst the 219 ginsenosides identified from PNF, only 40 prototype compounds together with 11 metabolites were characterized from the rat plasma, which could indicate a large proportion of PNF components were directly excreted by feces (direct evidence was not available in the current work). The 11 metabolites characterized in the plasma of rats within 3 h after oral administration were mainly the deglycation metabolites of the PPD-type ginsenosides, characterized by the elimination of Glc, Ara, and Xyl, etc., at C-3 and C-20. For instance, noto-Fh1 could lose the Xyl at C-3 or C-20 and thus be converted to **M3**. Some of them were the PPT-type ginsenoside deglycation metabolites and oxidation metabolites, such as the oxidation of Re to form **M1**, and the loss of Glc at C-20 for Rg2. A few of them were the OA-type ginsenoside deglycation metabolites. For example, Ro could lose one Glc at C-3 or C-28, transforms into **M6**, tentatively characterized as zingibroside R1.

#### 2.3.3. Isomer Verification Based on CCS Prediction 

Although MS is very powerful in the rapid characterization of herbal components as well as their in vivo metabolites, in most cases, the structures of compounds fail to be completely determined based on the fragmentation information alone. More dimension of available structure information may assist in enhancing the identification of isomeric metabolites, such as IM-derived CCS [44]. The HDMS^E^ approach enabled IM separation of all precursor ions based on the difference in charge state, size, and shape, generating the useful CCS value for each component.

**Figure 7:**
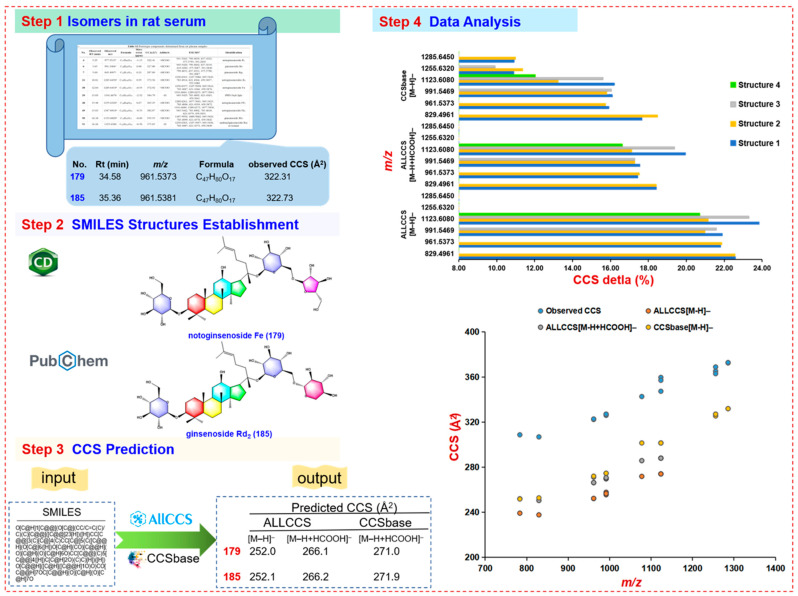
CCS prediction of ginsenoside isomers in rat plasma by ALLCCS and CCSbase. Step 1 to step 3: the isomer prediction and matching strategy based on ALLCCS and CCSbase; step 4: the selected *m/z* values and their CCS prediction and matching errors. Those selected ginsenoside isomers are annotated: *m/z* 829.4961 (**202#**-F2; **M5**-Rg2), *m/z* 961.5373 (**179#**-noto-Fe; **185#**-Rd2), *m/z* 991.5469 (**6#**-Re; **M8**-gypenoside XVII; **M7**-Rd), *m/z* 1123.6080 (**84#**-Rb2; **90#**-Rb3; **97#**-noto-L; **M4**-vina-R7), *m/z* 1255.6320 (**48#**-noto-FP2; **61#**-noto-Fc; **66#**-Ra1), *m/z* 1285.6450 (**31#**-noto-R4; **38#**-noto-Fa).

Using ALLCCS (http://allccs.zhulab.cn, accessed on 17 October 2022) and CCSbase (https://CCSbase.net, accessed on 17 October 2022) databases, we selected six groups of isomers (corresponding to 16 compounds in total) in rat plasma for CCS prediction. Among them, eleven compounds were identified by comparison with the reference compounds, as shown in Table S5 from Supplementary Materials. Detailed CCS prediction strategies and results are illustrated in Figure 7. Taking the prototype component **179#**-notoginsenoside Fe (*t*_R_, 34.58 min; C_47_H_80_O_17_) and **185#**-ginsenoside Rd_2_ (*t*_R_, 35.36 min; C_47_H_80_O_17_) as an example, the predicted CCS values of the [M–H]^−^ precursor ions by ALLCCS were 252.0 and 252.1, and those of the [M–H+HCOOH]^−^ precursor ions were 266.1 and 266.2, respectively. The CCS values of the [M–H]^−^ precursor ions predicted by CCSbase were 271.0 and 271.9, respectively. There was large difference between the predicted and the measured CCS values for **179#** and **185#**. We also found that the CCS values predicted by CCSbase for the isomers were very similar. Despite the ALLCCS prediction results can distinguish some isomers, but the database collects very limited ginsenosides, and the CCS delta (%) was relatively large. On the whole, the prediction error of ALLCCS was larger than that of CCSbase, and the CCS delta (%) of isomers was more than 16.00%. Studies had shown that, with the complex chemical structure, the CCS prediction error of metabolites with higher *m/z* tended to be larger [45], which is consistent with the results predicted by ALLCCS. Therefore, we might ascribe the large number of predicted CCS errors to the relatively large molecular mass of saponins. Since there are very few saponins in the database, the accuracy of this strategy needs to be further verified.

## 3. Materials and Methods

### 3.1. Chemicals and Materials

The 51 reference compounds of ginsenosides (Table S1 from Supplementary Materials), including 20(S)-ginsenosides F2, -F3, F4, -F5, -Rb1, -Rb2, -Rb3, -Ra1, -Ra2, -Ra3, -Rc, -Rd, -Re, -Rf, -Rd2, -Rh3, -Rk1, -Rk3, -Ro, -Rs3, -Rh1, Rh2, -Rg1, -Rg2, -Rg3, -F1, 20(R)-ginsenosides Rh1, -Rh2, -Rg2, -Rg3, 20(S)-notoginsenosides Fa, -Fc, -Fd, -Fe, -FP2, -Ft1, -S, -Fh1, -R1, R2, -R4, 20(R)-notoginsenoside R2, malonyl-ginsenosides Rb1, -Rb2, -Rd, -Rc, gypenoside XVII, vinaginsenoside R4, compound K, chikusetsusaponins L5, and -Iva were purchased from Shanghai Standard Biotech Co., Ltd. (Shanghai, China), or isolated from the flower buds of *P. notoginseng* by the authors. The purities for these 51 reference standards were higher than 95% determined by HPLC-UV. Raw materials of PNF were purchased from Shilin (Yunnan, China), and authenticated by Prof. Xian-kuan Li (Tianjin University of Tradition Chinese Medicine). Voucher specimen (No. PNF-20200912) was deposited in the author’s laboratory. Acetonitrile (Merck, Darmstadt, Germany) and formic acid (ACS, Wilmington, NC, USA) were both the HPLC grade. Ultra-pure water was prepared using a Millipore Alpha-Q Water purification system (Millipore, Bedford, MA, USA).

### 3.2. Sample Preparation

5 kg of dried PNF were cut into pieces and extracted twice with 50 L of a 70% (*v/v*) ethanol solution using ultrasound assistance (power: 1000 W; frequency: 40 kHz), each extraction taking two hours. The water bath temperature was kept below 40 °C. The two extracts were concentrated under reduced pressure to no alcohol flavor and freeze-dried to obtain the extract powder of PNF. Precision-weighed 5 mg of *Panax notoginseng* flower extract powder, adding 1 mL 70% (*v/v*) methanol solution to dissolve, vortexed fully. Took 200 µL, add 800 µL 70% (*v/v*) methanol solution, and vortex fully to obtain 1 mg/mL sample solution. After centrifugation at 21,912.8 xg (14,000 rpm) for 10 min at 4 °C, the supernatant was taken as the test solution.

For the multi-component and in vivo metabolite characterization, each of 51 reference compounds of ginsenosides was accurately weighed with 1 mg and dissolved in 1 mL 70% (*v/v*) methanol solution to prepare the standard solution with the concentration of 1 mg/mL. According to the principle of isomers and co-elution component in different groups, 17 reference compounds solutions were mixed into a group, and each reference compound solution was taken 50 µL to obtain 3 groups of mixed solutions, the concentration of each group of mixed solution was about 50 µg/mL.

### 3.3. Animal Experiments and the Preparation of Samples

Male Wistar rats (weighing 200 ± 20 g) were provided by the Weitong Lihua Animal Technology Co., Ltd. (Beijing, China). The rats were allowed to acclimatize for 4 days in an animal room and fed with the standard laboratory water and food. The animal breeding room was maintained at 22–24 °C and relative humidity of 60%. All animal experiments were performed in accordance with Institutional Animal Research Committee guidelines.

Twelve rats were randomly divided into four groups, including the blank group, high-dose group, middle-dose group, and low-dose group (*n* = 3). The extract of PNF was dissolved in water and then administrated orally to rats. In order to effectively detect the blood entering components in rat plasma, on the premise of the minimum lethal dose of PNF saponins in rats is not clear, we set three dose groups on the basis of the effective dose in hypertensive rats [46,47] and extraction rate of PNF total saponins. The low-dose group, middle-dose group and high-dose group were, respectively, administered 0.4 g/kg, 1.2 g/kg, and 3.6 g/kg (crude drug per g/kg of body weight) of PNF extract once a day, while the rats in the blank group were given the same volume of normal saline. The drug solution was continuously administered for 3 days. Rats were fasted for 12 h with the free access to water before the last administration. At 15, 30, 60, and 180 min after the last administration, 0.4 mL of blood was collected from the orbital venous plexus of the rat, respectively. Plasma samples were immediately centrifuged at 21,912.8× *g* (14,000 rpm) for 10 min at 4 °C. All the samples were stored at −80 °C until the analysis.

For identifying more prototype compounds and the transformed metabolites, the plasma samples collected at each time point were combined. Protein precipitation was conducted with methanol. An aliquot of 200 μL of the plasma sample was added with 1.2 mL of methanol, vortex mixed for 10 min, and then centrifuged at 21,912.8× *g* (14,000 rpm) for 10 min at 4 °C. The resultant supernatant was transferred into another tube and then evaporated to dryness under vacuum at 37 °C. The residue was redissolved in 200 μL of 70% methanol with the aid of vortex mixing. All the samples suffered from another centrifugation at 21,912.8× *g* (14,000 rpm) for 10 min at 4 °C as the test solution.

### 3.4. UHPLC/IM-QTOF-MS

In both the multicomponent characterization of PNF and identification of its metabolites in rat plasma, an ACQUITY UPLC I-Class/Vion^TM^ IM-QTOF hybrid high-resolution LC-MS system (Waters, Milford, MA, USA) was employed. A Waters HSS T3 column (100 mm × 2.1 mm, 1.8 μm) was used. The optimized parameters were set as follows: oven temperature, 40 °C; flow rate, 0.3 mL/min; injection volume, 2 μL; autosampler temperature, 4 °C. Solvent A (water containing 0.1% formic acid, v/v) and solvent B (acetonitrile containing 0.1% formic acid, *v/v*) were used as the mobile phase according to the gradient elution program as the follows: 15–20% B at 0–2 min; 20–30% B at 2–5 min; 30% B at 5–16 min; 30–34% B at 16–31 min; 34–65% B at 31–41 min; 65–95% B at 41–43 min); and 95% B at 43–45 min. When collecting the rat plasma data, the initial eluate with 0.8 min was automatedly switched into the waste to avoid contamination on the column.

The HDMS^E^ (PNF extract and rat plasma) and DDA (PNF extract) data were recorded on a Vion IM-QTOF mass spectrometer in the negative ESI mode with conditions set as follows: scan range, *m/z* 350–1500; capillary voltage, 1.0 kV; cone voltage, 20 V; source offset, 80 V; source temperature, 120 °C; desolvation temperature, 500 °C; desolvation gas flow rate (N^2^), 800 L/h; and cone gas flow rate (N^2^), 50 L/h. For the traveling wave IM separation, default parameters were defined [48]. In HDMS^E^, Low energy at 6 eV was set to record the information of all precursors, and high energy ramp of 40–80 eV was predefined to acquire the CID fragmentation information of all recorded precursors. In the case of DDA, the same low energy at 6 eV was set to record the information of precursors for full scan, while mass dependent ramp collision energy (MDRCE) of 10–35 eV for low mass and 65–90 eV for high mass was set to automatedly acquire the fragmentation information of top three most intense precursors in consistency with the criteria as follows: When TIC (total ion chromatogram) intensity exceeded 1000 detector counts, the MS/MS fragmentation of three most intense precursors was automatically triggered at 0.2 s per scan over a mass range of *m/z* 350–1500. The MS/MS acquisition stopped if time exceeded 0.7 s. For HDMS^E^ and DDA data calibration, the leucine enkephalin solution (at 200 ng/mL; Sigma-Aldrich, St. Louis, MO, USA) was constantly infused at 10 μL/min.

### 3.5. Automated Peak Annotation Workflows Facilitated by UNIFI^TM^ and Searching an In-House Ginsenoside Library Enabling the Efficient Multicomponent Characterization of PNF

Automated workflows for processing the HDMS^E^ and DDA data (e.g., data correction, peak extraction, and peak annotation) in the efficient multicomponent characterization of PNF were established by the UNIFI^TM^ software (Waters, Milford, MA, USA) via searching against an inhouse ginsenoside library, which had 573 known ginsenosides recorded [45]. Firstly, an inhouse library, including the compound name, chemical formula, and molecular weight information of components, was established through searching the web databases including PubMed (http://pubchem.ncbi.nlm.nih.gov, accessed on 17 December 2020), Web of Science (www.webofknowledge.com, accessed on 17 December 2020), Chinese National Knowledge Infrastructure (CNKI, www.cnki.net, accessed on 17 December 2020), and Traditional Chinese Medicines Integrated Database (TCMID, http://www.megabionet.org/tcmid/, accessed on 17 December 2022). The established table was directly imported into UNIFI. Secondly, the DDA and HDMS^E^ data were processed by the established data processing method, which generated a list of the characterized components as “Identified Components”, including various information such as the observed *m/z*, formula, observed t_R_, mass error, adducts, etc. Various approaches (e.g., adduct filtering for carboxyl-free neutral ginsenosides, removing false positives that result from adducts or in-source fragmentation ions, removing invalid data with duplicates, similar retention time, and incomplete fragment information) were utilized to confirm the reliability of the identification results and discriminate false positives. For the list of the “Unknown Components”, the components were sorted by removing data with duplicates, similar retention time, and response ≤10,000, to enrich the identification results based on the high-accuracy MS^1^ and MS^2^ data. The CCS value in the HDMS^E^ data was added to the DDA identification result. The two identification lists were combined to obtain the final identification result. The processing parameters were set as follows. Retention time range, 0–45.0 min. Find 4D Peaks (only set in HDMS^E^): High-energy intensity threshold, 500.0 counts; low-energy intensity threshold, 1000.0 counts. DDA: MS ion intensity threshold, 1000.0 counts; MS/MS ion strength threshold: 500.0 counts. Target by mass: target match tolerance, 10.0 ppm; screen on all isotopes in a candidate, generate predicted fragments from structure, and look for in-source fragments were enabled; fragment match tolerance, 10.0 ppm. Adducts: [M–H]^–^, [M–H+HCOOH]^–^ in the negative mode. Lock mass: combine width, 3 scans; mass window, 0.5 *m/z*; reference mass, leucine encephalin (at *m/z* 554.2620); reference charge, –1.

### 3.6. Identification of the Prototype Compounds and In Vivo Metabolites in Rat Plasma Based on UNIFI^TM^ Platform

The raw data were processed on the automatic peak annotation and Met-ID function of UNIFI platform to achieve the reliable and systematic identification of the multiple characterization of the metabolites of PNF after oral administration in rat plasma [49,50]. The processing parameters of the pathway-profiling IMS mode were set as follows: the selection of adducts, [M–H]^−^ and [M–H + HCOOH]^−^ in negative mode; retention time range, 0.8–45.0 min; target mass match tolerance, 10.0 ppm; filter type, cluster; binary comparison, relative intensity threshold, 30%. For lock mass, leucine encephalin (at *m/z* 554.2620) was used to perform the data calibration in the negative ESI mode, with combine width of 3 scans and mass window of 0.5 *m/z*. The UNIFI pathway metabolite prediction function is used for data processing and analysis: input the chemical structure of the prototype component, submit prediction according to the possible metabolic situation of the prototype component or the reported ginsenosides and set the possible metabolic reaction of the compound. A list of potential biotransformation rules expected was employed as follows: oxidation, dehydration, hydration, glucuronidation, methylation, sulfation, decarboxylation, glutathione conjugation, cysteine conjugation, and acetylcysteine conjugation. Select blank sample as control reference, edit sample information and processed data.

Select dosed group data and blank group data, the software generates the predicted metabolite identification list of the prototype component after processing chromatographic peak identification, deconvolution, and extraction of chromatographic peak information, which include molecular formula, retention time, *m/z*, response intensity, mass deviation, metabolic reaction pathway, and other information. False positive results and invalid data can be excluded by deduplicating and deleting ions with large mass deviation value and incomplete fragment information. Data can be deeply mined, and fragment information confirmed by combining MDF, NLF, and CFS. By comparing the chromatograms of PNF-treated rat plasma with those of blank group, the prototype constituents and metabolites were identified to achieve a comprehensive characterization of in vivo metabolites in the blood samples, recording the retention time of metabolites, *m/z*, MS/MS fragments and other information, and derivation of metabolic pathways.

## 4. Conclusions

In the current work, a powerful UHPLC/IM-QTOF-MS approach was established, enabling the comprehensive multicomponent characterization of PNF and the in vivo metabolites in rat plasma after gastric perfusion of PNF extract. The integration of DDA and HDMS^E^ exerted complementarity in characterizing PNF components, allowing a total of 219 saponins to be unambiguously identified or tentatively characterized Meanwhile, we identified 51 components, including 40 prototype components and 11 metabolites in rat plasma by using the Met-ID function of UNIFI. The metabolic pathways of PNR saponins in rats mainly included phase I reactions (e.g., deglycosylation and oxidation). To the best of our knowledge, it is the first report on the in vivo metabolism of PNF. The results obtained in this study can help in better understanding the effective material basis of PNF, beneficial to the quality control of PNF and *P. notoginseng*. Moreover, the analytical strategy we utilized in this work could be an example for characterizing the chemical components and in vivo metabolites of the other TCM.

## Supplementary Materials

The following supporting information can be downloaded at: https://www.mdpi.com/article/10.3390/molecules27249049/s1, Figure S1. Stationary phase screening for the establishment of UPHLC/IM-QTOF-MS by showing the base peak chromatogram (BPCs) of PNF extract on 20 candidate reversed-phase chromatographic columns; Figure S2. Histograms of the numbers of detected components under three different top N settings. The data were obtained by processing the negative HDMS^E^ data using UNIFI, generating the lists of “Identified Components” and “Unknown Components”; Figure S3 MS/MS spectra of 18 prototype components of PNF-administrated rat plasma; Table S1. Information for 51 ginsenoside reference compounds used in this work; Table S2. Information for the 219 saponins characterized from the flower bud of *Panax notoginseng*; Table S3. Information for the 40 prototype components characterized from rat plasma; Table S4. Information for 11 metabolites identified from rat plasma; Table S5. CCS prediction of isomers in rat plasma based on ALLCCS and CCSbase.
