# Supplementary material for: Multicomponent Characterization of the Flower Bud of Panax notoginseng and Its Metabolites in Rat Plasma by Ultra-High Performance Liquid Chromatography/Ion Mobility Quadrupole Time-of-Flight Mass Spectrometry
Source: Molecules. 2022 Dec 19;27(24):9049. doi: 10.3390/molecules27249049 (PMC9786607; doi:10.3390/molecules27249049)
Supplement: Supplementary file 1 [file molecules-27-09049-s001.zip › molecules-2105537-supplementary.pdf]

molecules

MDPI

*Supplementary Materials*

# Multicomponent Characterization of the Flower Bud of *Panax notoginseng* and Its Metabolites in Rat Plasma by Ultra-High Performance Liquid Chromatography/Ion Mobility Quadrupole Time-of-Flight Mass Spectrometry

**Xiaonan Yang [1,2,†], Ying Xiong [1,2,†], Hongda Wang [1,2,†], Meiting Jiang [1,2], Xiaoyan Xu [1,2], Yueguang Mi [1,2], Jia Lou [1,2], Xiaohang Li [1,2], He Sun [1,2], Yuying Zhao [1,2], Xue Li [1,2,*] and Wenzhi Yang [1,2,*]**

[1] State Key Laboratory of Component-based Chinese Medicine, Tianjin University of Traditional Chinese Medicine, 10 Poyanghu Road, , Tianjin 301617, China
[2] Haihe Laboratory of Modern Chinese Medicine, Tianjin University of Traditional Chinese Medicine, 10 Poyanghu Road, Tianjin 301617, China
* Correspondence: tjdxsyx@163.com (X.L.); wzyang0504@tjutcm.edu.cn (W.Y.); Tel.: +86-022-5979-1833 (W.Y.)
† These authors contributed equally to this work.

Contents

Figure S1 Stationary phase screening for the establishment of UPHLC/IM-QTOF-MS by showing the base peak chromatogram (BPCs) of PNF extract on 20 candidate reversed-phase chromatographic columns.

Figure S2 Histograms of the numbers of detected components under three different top N settings. The data were obtained by processing the negative HDMS$^E$ data using UNIFI, generating the lists of "Identified Components" and "Unknown Components".

Figure S3 MS/MS spectra of 18 prototype components of PNF-administrated rat plasma.

Table S1 Information for 51 ginsenoside reference compounds used in this work.

Table S2 Information for the 219 saponins characterized from the flower bud of *Panax notoginseng*.

Table S3 Information for the 40 prototype components characterized from rat plasma.

Table S4 Information for 11 metabolites identified from rat plasma.

Table S5 CCS prediction of isomers in rat plasma based on ALLCCS and CCSbase.

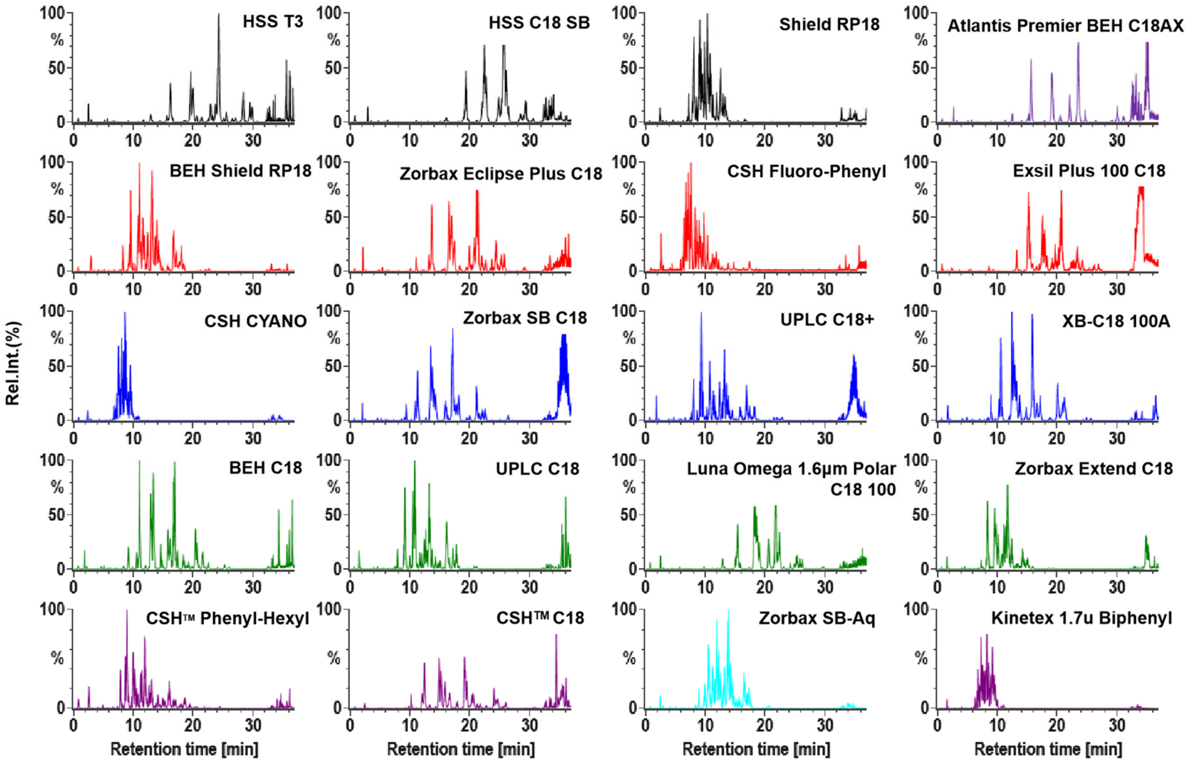


**Figure S1.** Stationary phase screening for the establishment of UPHLC/IM-QTOF-MS by showing the base peak chromatogram (BPCs) of PNF extract on 20 candidate reversed-phase chromatographic columns.

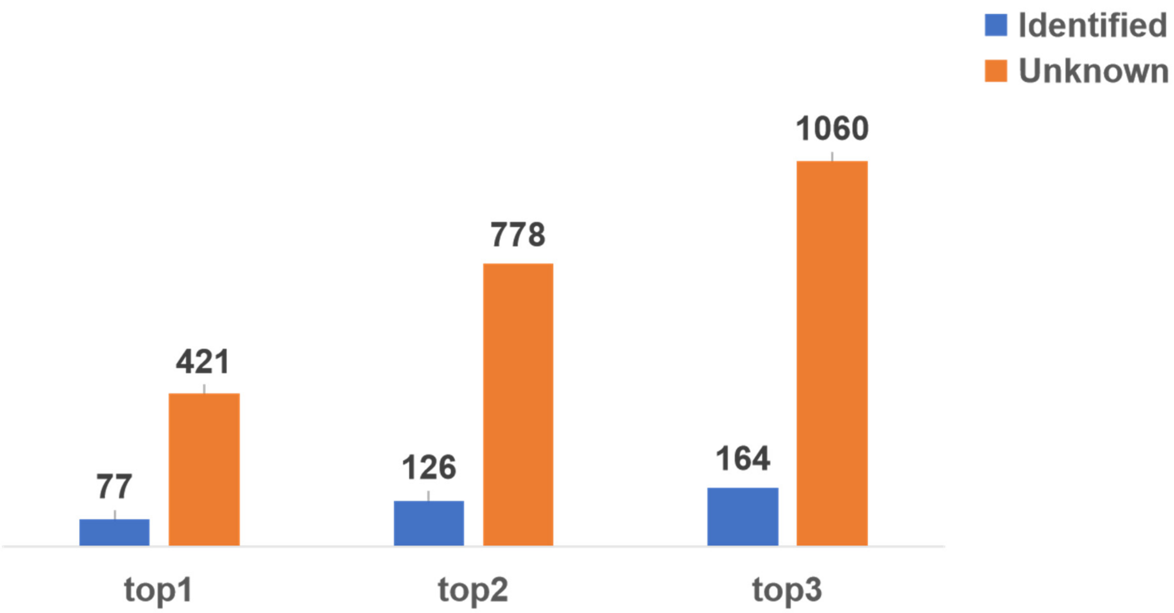


**Figure S2.** Histograms of the numbers of detected components under three different top N settings. The data were obtained by processing the negative $HDMS^E$ data using UNIFI, generating the lists of "Identified Components" and "Unknown Components".

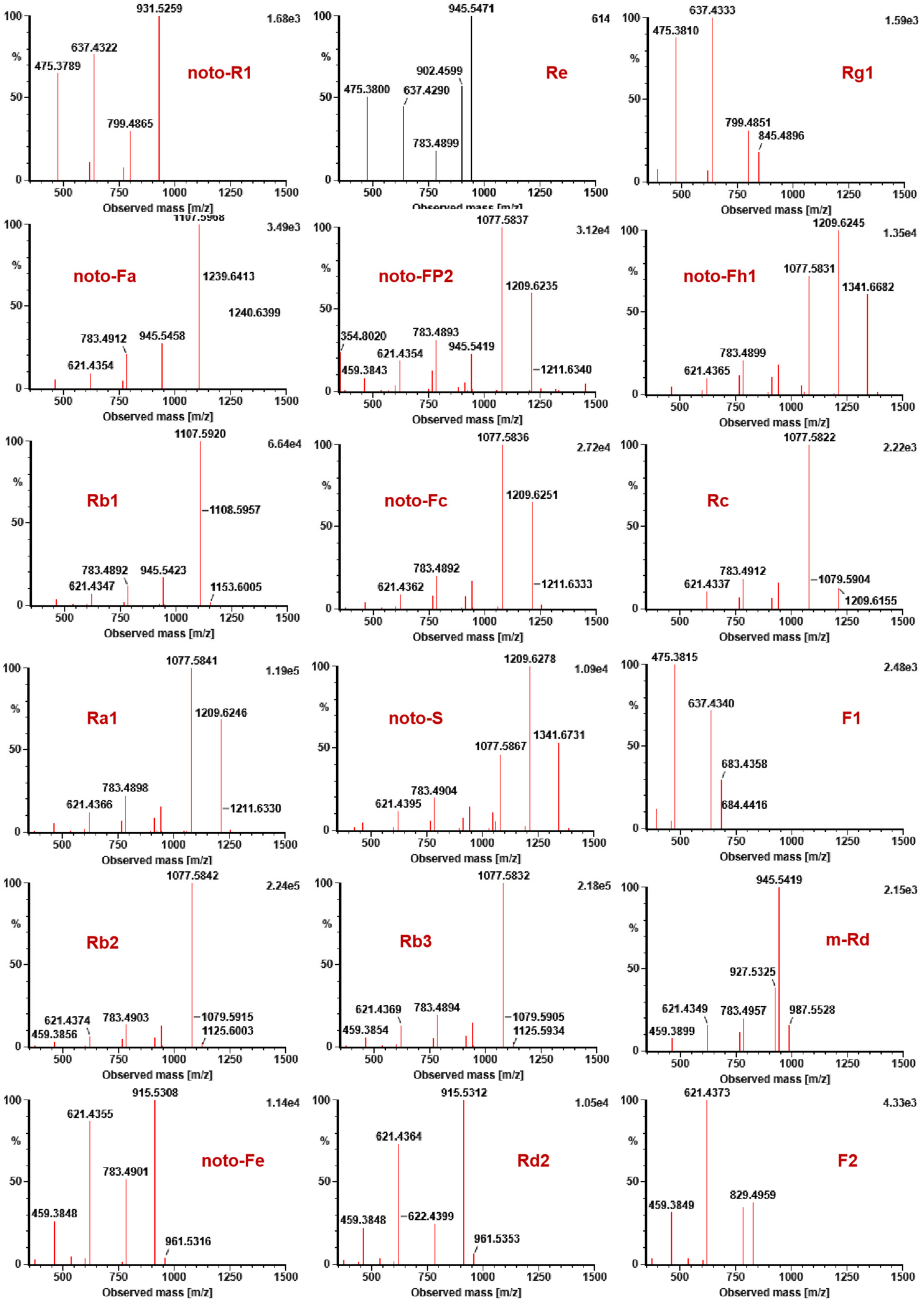


**Figure S3.** MS/MS spectra of 18 prototype components of PNF-administrated rat plasma. (Noto-R4, Ro, and m-Rb1 failed to provide MS/MS spectra due to low response).

**Table S1.** Information for 51 ginsenoside reference compounds used in this work.

| No. | Trivial name | M.F. | Exact Mass | Subclass |
|---|---|---|---|---|
| **1** | ginsenoside F2 | $C_{42}H_{72}O_{13}$ | 784.4901 | PPD |
| **2** | ginsenoside Rb2 | $C_{53}H_{90}O_{22}$ | 1078.5924 | |
| **3** | ginsenoside Rb1 | $C_{54}H_{92}O_{23}$ | 1108.6029 | |
| **4** | ginsenoside Ra1 | $C_{58}H_{98}O_{26}$ | 1210.6346 | |
| **5** | ginsenoside Rd | $C_{48}H_{82}O_{18}$ | 946.5501 | |
| **6** | 20(*S*)-ginsenoside Rh2 | $C_{36}H_{62}O_{8}$ | 622.4445 | |
| **7** | 20(*R*)-ginsenoside Rh2 | $C_{36}H_{62}O_{8}$ | 622.4445 | |
| **8** | notoginsenoside Fc | $C_{58}H_{98}O_{26}$ | 1210.4346 | |
| **9** | notoginsenoside R4 | $C_{59}H_{100}O_{27}$ | 1240.6452 | |
| **10** | notoginsenoside Ft1 | $C_{47}H_{80}O_{17}$ | 916.5396 | |
| **11** | ginsenoside Rb3 | $C_{53}H_{90}O_{22}$ | 1078.5924 | |
| **12** | ginsenoside Ra2 | $C_{58}H_{98}O_{26}$ | 1210.6346 | |
| **13** | 20(*S*)-ginsenoside Rg3 | $C_{42}H_{72}O_{13}$ | 784.4973 | |
| **14** | 20(*R*)-ginsenoside Rg3 | $C_{42}H_{72}O_{13}$ | 784.4973 | |
| **15** | notoginsenoside Fe | $C_{47}H_{80}O_{17}$ | 916.5396 | |
| **16** | notoginsenoside Fa | $C_{59}H_{100}O_{27}$ | 1240.6452 | |
| **17** | ginsenoside Rs3 | $C_{44}H_{74}O_{14}$ | 826.5079 | |
| **18** | ginsenoside Rc | $C_{53}H_{90}O_{22}$ | 1078.5924 | |
| **19** | compound K | $C_{36}H_{62}O_{8}$ | 622.4445 | |
| **20** | notoginsenoside FP2 | $C_{58}H_{98}O_{26}$ | 1210.6346 | |
| **21** | notoginsenoside Fd | $C_{47}H_{80}O_{17}$ | 916.5396 | |
| **22** | notoginsenoside S | $C_{63}H_{106}O_{30}$ | 1342.6769 | |
| **23** | notoginsenoside Fh1 | $C_{63}H_{106}O_{30}$ | 1342.6769 | |
| **24** | ginsenoside Ra3 | $C_{59}H_{100}O_{27}$ | 1240.6452 | |
| **25** | gypenoside XVII | $C_{48}H_{82}O_{18}$ | 946.5501 | |
| **26** | ginsenoside Rd2 | $C_{47}H_{80}O_{17}$ | 916.5396 | |
| **27** | ginsenoside F3 | $C_{41}H_{70}O_{13}$ | 770.4816 | PPT |
| **28** | notoginsenoside R1 | $C_{47}H_{80}O_{18}$ | 932.5345 | |
| **29** | 20(*R*)-notoginsenoside R2 | $C_{41}H_{70}O_{13}$ | 770.4816 | |
| **30** | 20(*S*)-notoginsenoside R2 | $C_{41}H_{70}O_{13}$ | 770.4816 | |
| **31** | 20(*R*)-ginsenoside Rh1 | $C_{36}H_{62}O_{9}$ | 638.4394 | |
| **32** | 20(*S*)-ginsenoside Rh1 | $C_{36}H_{62}O_{9}$ | 638.4394 | |
| **33** | 20(*R*)-ginsenoside Rg2 | $C_{42}H_{72}O_{13}$ | 784.4973 | |
| **34** | 20(*S*)-ginsenoside Rg2 | $C_{42}H_{72}O_{13}$ | 784.4973 | |
| **35** | ginsenoside Rg1 | $C_{42}H_{72}O_{14}$ | 800.4922 | |
| **36** | vinaginsenoside R4 | $C_{48}H_{82}O_{19}$ | 962.5450 | |
| **37** | ginsenoside F5 | $C_{41}H_{70}O_{13}$ | 770.4816 | |

| | | | | |
|---|---|---|---|---|
| **38** | ginsenoside Rf | $C_{42}H_{72}O_{14}$ | 800.4922 | |
| **39** | ginsenoside Re | $C_{48}H_{82}O_{18}$ | 946.5501 | |
| **40** | chikusetsusaponin L5 | $C_{46}H_{78}O_{17}$ | 902.5239 | |
| **41** | 20(*S*)-ginsenoside F1 | $C_{36}H_{62}O_{9}$ | 638.4394 | |
| **42** | ginsenoside F4 | $C_{42}H_{70}O_{12}$ | 766.4867 | Other |
| **43** | ginsenoside Rh3 | $C_{36}H_{60}O_{7}$ | 604.4339 | |
| **44** | ginsenoside Rk1 | $C_{42}H_{70}O_{12}$ | 766.4867 | |
| **45** | ginsenoside Rk3 | $C_{36}H_{60}O_{8}$ | 620.4288 | |
| **46** | malonyl-ginsenoside Rb1 | $C_{57}H_{94}O_{26}$ | 1194.6033 | Mal |
| **47** | malonyl-ginsenoside Rb2 | $C_{56}H_{92}O_{25}$ | 1164.5928 | |
| **48** | malonyl-ginsenoside Rd | $C_{51}H_{84}O_{21}$ | 1032.5505 | |
| **49** | malonyl-ginsenoside Rc | $C_{56}H_{92}O_{25}$ | 1164.5928 | |
| **50** | ginsenoside Ro | $C_{48}H_{76}O_{19}$ | 956.4981 | OA |
| **51** | chikusetsusaponin IVa | $C_{42}H_{66}O_{14}$ | 794.4453 | |

**Table S2.** Information for the 219 saponins characterized from the flower bud of *Panax notoginseng*.

| No. | Observed RT (min) | Observed m/z | Formula | Mass error (ppm) | CCA(Å$^2$) | Adducts | ESI-MS$^2$ | Identification |
|---|---|---|---|---|---|---|---|---|
| **1** | 4.95 | 977.5347[d/f] | $C_{47}H_{80}O_{18}$ | 2.09 | 314.48 | +HCOO | 931.5317, 799.4833, 637.4327, 475.3777 | quinquenoside L17 or isomer |
| **2** | 4.99 | 1109.5779[b/e/f] | $C_{53}H_{90}O_{24}$ | 2.66 | 331.14 | +HCOO | 1063.5673, 931.5302, 637.4337, 475.3743 | PPT-xyl-2rha-glc |
| **3** | 5.04 | 1007.5459[d/f] | $C_{48}H_{82}O_{19}$ | −2.34 | 324.85 | +HCOO | 961.5355, 799.4810, 637.4307, 475.3768 | chikusetsusaponin LM4 or isomer |
| **4** | 5.28 | 977.5315[a] | $C_{47}H_{80}O_{18}$ | −1.15 | 322.41 | +HCOO | 931.5263, 799.4839, 637.4322, 475.3793, 391.2845 | notoginsenoside R1 |
| **5** | 5.67 | 945.5432[c/f] | $C_{48}H_{82}O_{18}$ | 0.38 | 324.43 | −H | 799.4837, 637.4319, 475.3791, 391.2850 | ginsenoside Rg18 or isomer |
| **6** | 5.68 | 991.5484[a] | $C_{48}H_{82}O_{18}$ | 0.06 | 327.00 | +HCOO | 945.5429, 783.4936, 637.4319, 619.4202, 475.3887, 391.2848 | ginsenoside Re |
| **7** | 5.73 | 845.4997[a] | $C_{42}H_{72}O_{14}$ | 0.21 | 297.89 | +HCOO | 799.4851, 637.4321, 475.3798, 391.2867 | ginsenoside Rg1 |
| **8** | 6.01 | 1025.5559[d/f] | $C_{48}H_{84}O_{20}$ | 1.00 | 334.87 | +HCOO | 979.5493, 817.4986, 655.4431, 493.3887, 475.3794 | ginsengenin-S3 or isomer |
| **9** | 6.10 | 885.4862[b/e/f] | $C_{48}H_{72}O_{13}$ | −4.46 | 303.56 | −H | 885.4840, 799.4862, 637.4324, 475.3806, 391.2857 | PPT-mal-2glc |
| **10** | 6.32 | 1139.5858[b/e/f] | $C_{54}H_{92}O_{25}$ | 0.26 | 359.88 | +HCOO | 1093.5788, 961.5443, 799.4830, 637.4346, 475.3806 | PPT-xyl-3glc |
| **11** | 6.42 | 1271.6292[d/f] | $C_{58}H_{98}O_{27}$ | 2.06 | 359.99 | +HCOO | 1225.6248, 1093.5824, 961.5395, 799.4849, 637.4314, 475.3782 | notoginsenosides NL-B3 or isomer |
| **12** | 6.48 | 1169.5967[d/f] | $C_{54}H_{92}O_{24}$ | 2.87 | 356.15 | +HCOO | 1123.5938, 961.5368, 799.4857, 637.4347, 475.3740 | 6-*O*-[β-*D*-glucopyranosyl-(1→2)-β-*D*-glucopyranosyl]-20-*O*-[β-*D*-glucopyranosyl-(1→4)-β-*D*-glucopyranosyl]-20(*S*)-protopanaxatriol or isomer |
| **13** | 6.72 | 1139.5865[b/f] | $C_{54}H_{92}O_{25}$ | 0.88 | 365.09 | +HCOO | 1093.5801, 961.5363, 799.4864, 781.4749, 637.4324, 475.3803 | PPT-xyl-3glc |
| **14** | 6.99 | 1209.6048[b/e/f] | $C_{61}H_{94}O_{24}$ | −1.18 | 342.71 | −H | 1123.5899, 961.5276, 799.4836, 637.4332, 475.3776 | PPT-mal-4glc |
| **15** | 7.13 | 1139.5868[b/f] | $C_{53}H_{90}O_{23}$ | 1.17 | 355.51 | +HCOO | 1093.5806, 961.5403, 799.4856, 637.4334, 475.3791 | PPT-xyl-3glc |
| **16** | 7.37 | 1179.5825[b/e/f] | $C_{56}H_{92}O_{26}$ | 1.77 | 347.77 | -H | 1093.5811, 961.5401, 799.4880, 637.4314, 475.3809 | PPT-mal-xyl-3glc |
| **17** | 7.67 | 1155.5809[b/e/f] | $C_{53}H_{90}O_{24}$ | −2.73 | 352.66 | +HCOO | 1109.5719, 1091.5697, 797.4728, 635.4145, 473.3642, 455.3563 | $C_{30}H_{48}O_3$-$H_2O$-xyl-3glc |
| **18** | 7.70 | 1139.5885[b/f] | $C_{53}H_{90}O_{23}$ | 2.82 | 360.70 | +HCOO | 1093.5831, 961.5359, 799.4851, 637.4339, 475.3794 | PPT-xyl-3glc |
| **19** | 7.81 | 1179.5829[b/e/f] | $C_{56}H_{92}O_{26}$ | 2.11 | 343.99 | −H | 1093.5828, 961.5361, 799.4853, 781.4738, 637.4317, 475.3796 | PPT-mal-xyl-3glc |

| | | | | | | | | |
|---|---|---|---|---|---|---|---|---|
| **20** | 7.93 | 1007.5573[a] | $C_{48}H_{82}O_{19}$ | 0.26 | 332.65 | +HCOO | 961.5380, 799.4842, 637.4299, 475.3807 | vina ginsenoside $R_4$ |
| **21** | 8.21 | 1179.5815[b/e/f] | $C_{56}H_{92}O_{26}$ | 0.93 | 348.65 | –H | 1093.5801, 961.5381, 799.4886, 781.4746, 637.4322, 475.3796 | PPT-mal-xyl-3glc |
| **22** | 8.62 | 1179.5824[b/e/f] | $C_{56}H_{92}O_{26}$ | 1.69 | 346.23 | –H | 1093.5826, 961.5357, 799.4850, 781.4749, 637.4304, 475.3789 | PPT-mal-xyl-3glc |
| **23** | 8.94 | 1195.5739[b/e/f] | $C_{56}H_{92}O_{27}$ | –1.19 | 350.69 | –H | 1109.5717, 1091.5532, 797.4655, 635.4175, 617.4071, 455.3482 | $C_{30}H_{48}O_3$-mal-$2H_2O$-xyl-3glc |
| **24** | 9.04 | 1137.5712[b/f] | $C_{53}H_{88}O_{23}$ | 0.77 | 355.38 | +HCOO | 1091.5652, 959.5242, 797.4716, 635.4144, 617.4066, 455.3552 | $C_{30}H_{48}O_3$-xyl-3glc |
| **25** | 9.14 | 1417.6852[b/f] | $C_{64}H_{108}O_{31}$ | –0.50 | 396.96 | +HCOO | 1371.6795, 1239.6379, 1107.5963, 945.5434, 783.4911, 621.4374, 459.3847, 353.1078 | PPD-2xyl-4glc |
| **26** | 9.28 | 1049.5527[d/f] | $C_{50}H_{84}O_{20}$ | –3.20 | 344.20 | +HCOO | 1003.5451, 961.5396, 799.4855, 637.4335, 475.3800, 391.2832 | 6-acetyl ginsenoside Rg3 or isomer |
| **27** | 10.03 | 947.5243[d/f] | $C_{47}H_{80}O_{19}$ | 2.36 | 311.48 | +HCOO | 901.5179, 769.4735, 637.4312, 475.3807 | notoginsenoside Rw1 or isomer |
| **28** | 10.18 | 845.4899[d/f] | $C_{42}H_{72}O_{14}$ | 0.09 | 301.66 | +HCOO | 799.4850, 637.4345, 491.3763 | 20(*R*)-pseudoginsenoside F11 or isomer |
| **29** | 10.43 | 947.5235[d/f] | $C_{46}H_{78}O_{17}$ | 3.30 | 308.38 | +HCOO | 901.5196, 769.4787, 475.3762 | chikusetsusaponin LM2 or isomer |
| **30** | 10.69 | 845.4912[d/f] | $C_{42}H_{72}O_{14}$ | 0.84 | 300.20 | +HCOO | 799.4856, 637.4346, 475.3800, 391.2863 | 20(*S*)-ginsenoside Rf or isomer |
| **31** | 10.86 | 1285.6450[a] | $C_{59}H_{100}O_{27}$ | 0.95 | 372.56 | +HCOO | 1239.6391, 1107.5986, 945.5443, 783.4914, 621.4364, 459.3857, 353.1086 | notoginsenosides R4 |
| **32** | 11.39 | 815.4801[a] | $C_{41}H_{70}O_{13}$ | 0.26 | 298.98 | +HCOO | 769.4732, 637.4328, 475.3802, 391.2842 | 20(*S*)-notoginsenoside R2 |
| **33** | 11.67 | 1387.6756[b/e/f] | $C_{63}H_{106}O_{30}$ | –0.16 | 383.00 | +HCOO | 1341.6694, 1209.6275, 1077.5866, 945.5441, 783.4914, 621.4379, 459.3848 | notoginsenoside Q or Fh1 (PPD-3xyl-3glc) |
| **34** | 11.70 | 947.5218[a] | $C_{46}H_{78}O_{17}$ | –0.34 | 321.67 | +HCOO | 901.5168, 769.4745, 637.4331, 475.3796, 391.2849 | chikusetsusaponin L5 |
| **35** | 11.88 | 1315.6529[d/f] | $C_{60}H_{102}O_{28}$ | 0.48 | 375.87 | +HCOO | 1269.6491, 1107.5946, 945.5464, 783.4926, 621.4363, 459.3794 | ginsenoside Rb5 or isomer |
| **36** | 12.02 | 1417.6849[b/f] | $C_{64}H_{108}O_{31}$ | 0.23 | 391.45 | +HCOO | 1371.6805, 1209.6280, 1077.5863, 945.5479, 783.4892, 621.4388, 459.3838 | PPD-4glc-2xyl |
| **37** | 12.26 | 1387.6772[b/e/f] | $C_{63}H_{106}O_{30}$ | –0.16 | 382.62 | +HCOO | 1341.6694, 1209.6283, 1077.5849, 945.5443, 783.4888, 621.4370, 459.3837 | PPD-3xyl-3glc |
| **38** | 12.92 | 1285.6412[a] | $C_{59}H_{100}O_{27}$ | –0.53 | 372.92 | +HCOO | 1239.6357, 1107.5938, 945.5416, 783.4887, 621.4366, 459.3876 | notoginsenoside Fa |
| **39** | 13.14 | 1341.6665[c/f] | $C_{63}H_{106}O_{30}$ | –2.32 | 384.79 | –H | 1341.6664, 1209.6253, 1077.5841, 945.5425, 783.4892, 621.4363, 459.3841 | PPD-3xyl-2glc |
| **40** | 13.31 | 829.4945[a] | $C_{42}H_{72}O_{13}$ | –1.21 | 302.67 | +HCOO | 783.4869, 637.4288, 475.3795 | 20(*S*)-ginsenoside Rg2 |
| **41** | 13.69 | 683.4361[a] | $C_{36}H_{62}O_{9}$ | –1.42 | – | +HCOO | 637.4312, 475.3790, 391.2845 | 20(*S*)-ginsenoside Rh1 |
| **42** | 14.17 | 1285.6450[d/f] | $C_{59}H_{100}O_{27}$ | 0.47 | 367.32 | +HCOO | 1239.6385, 1077.5860, 945.5416, 783.4900, 621.4380, 459.3840 | chikusetsusaponin VI or isomer |

| | | | | | | | | |
|---|---|---|---|---|---|---|---|---|
| **43** | 14.34 | 1255.6334[d/f] | $C_{58}H_{98}O_{26}$ | 0.45 | 357.40 | +HCOO | 1209.6279, 1077.5879, 945.5448, 783.4903, 621.4365, 459.3855 | notoginsenoside-FZ or isomer |
| **44** | 14.50 | 815.4802[a] | $C_{41}H_{70}O_{13}$ | 0.38 | 300.73 | +HCOO | 769.4739, 637.4308, 475.3801, 391.2863 | ginsenoside F3 |
| **45** | 14.90 | 1355.6619[b/e/f] | $C_{67}H_{104}O_{28}$ | −1.65 | 380.71 | −H | 1269.6495, 1107.5961, 945.5439, 783.4899, 621.4364, 459.3872 | PPD-mal-5glc |
| **46** | 15.03 | 1325.6562[b/f] | $C_{66}H_{102}O_{27}$ | 0.50 | 370.80 | −H | 1239.6386, 1107.5980, 945.5439, 783.4889, 621.4364, 459.3835 | PPD-mal-xyl-4glc |
| **47** | 15.18 | 1427.6713[b/e/f] | $C_{66}H_{108}O_{33}$ | 0.90 | 399.08 | −H | 1341.6725, 1209.6271, 1077.5861, 945.5463, 783.4899, 621.4379, 459.3838 | PPD-mal-3xyl-3glc |
| **48** | 15.56 | 1255.6315[a] | $C_{58}H_{98}O_{26}$ | 0.67 | 365.25 | +HCOO | 1209.6261, 1077.5841, 945.5425, 783.4896, 621.4359, 459.3872 | notoginsenoside FP2 |
| **49** | 15.84 | 1387.6914[a] | $C_{63}H_{106}O_{30}$ | −0.53 | 302.07 | +HCOO | 1341.6689, 1209.6272, 1077.5856, 945.5442, 783.4902, 765.4810, 621.4379, 459.3851 | notoginsenoside Fh1 |
| **50** | 16.18 | 1153.6010[a] | $C_{54}H_{92}O_{23}$ | −0.60 | 355.55 | +HCOO | 1107.5950, 1089.5862, 945.5424, 783.4899, 621.4378, 459.3842 | ginsenoside Rb1 |
| **51** | 16.47 | 1325.6375[b/f] | $C_{62}H_{102}O_{30}$ | −0.58 | 373.85 | −H | 1239.6383, 1107.5957, 945.5436, 783.4907, 621.4373, 459.3849 | PPD- mal-xyl-4glc |
| **52** | 16.58 | 1255.6320[d/f] | $C_{58}H_{98}O_{26}$ | 0.12 | 361.25 | +HCOO | 1209.6275, 1077.5861, 945.5440, 783.4904, 621.4373, 459.3847 | notoginsenoside-FZ or isomer |
| **53** | 17.01 | 1285.6456[d/f] | $C_{59}H_{100}O_{27}$ | 2.56 | 378.02 | +HCOO | 1239.6411, 1077.5892, 945.5443, 783.4880, 621.4335, 459.3828 | ginsenoside Ra3 or isomer |
| **54** | 17.38 | 1427.6714[b/e/f] | $C_{66}H_{108}O_{33}$ | 0.97 | 394.31 | −H | 1341.6744, 1323.6604, 1209.6280, 1077.5861, 915.5334, 783.4920, 621.4379, 459.3842 | PPD-mal-3xyl-3glc |
| **55** | 17.77 | 1295.6294[b/e/f] | $C_{61}H_{100}O_{29}$ | 1.27 | 364.76 | −H | 1209.6284, 1077.5880, 945.5444, 783.4899, 621.4377, 459.3847 | PPD-mal-2xyl-3glc |
| **56** | 17.83 | 955.4923[a] | $C_{48}H_{76}O_{19}$ | 1.54 | 200.20 | −H | 955.4915,793.4887,613.3763,569.3852,523.3783,497.3650,455.3536 | ginsenoside Ro |
| **57** | 17.93 | 855.4841[b/f] | $C_{48}H_{72}O_{13}$ | −6.91 | 294.69 | −H | 769.4763,475.3796 | PPT-mal-xyl-glc |
| **58** | 18.14 | 1285.6434[d/f] | $C_{59}H_{100}O_{27}$ | −0.10 | 376.64 | +HCOO | 1239.6378,1077.5868,945.5436,783.4904, 621.4361,459.3836 | chikusetsusaponin VI or isomer |
| **59** | 18.31 | 1193.5966[a] | $C_{57}H_{94}O_{26}$ | 0.49 | 265.46 | −H | 1107.5965,945.5437,783.4904,621.4378,459.3853 | malonyl-ginsenoside Rb1 |
| **60** | 18.50 | 1295.6264[b/e/f] | $C_{61}H_{100}O_{29}$ | −1.04 | 368.16 | −H | 1209.6282, 1077.5860, 945.5454, 783.4882, 621.4351, 459.3817 | PPD-mal-2xyl-3glc |
| **61** | 18.72 | 1255.6323[a] | $C_{58}H_{98}O_{26}$ | 0.80 | 368.97 | +HCOO | 1209.6268, 1077.5853, 945.5437, 783.4903, 621.4368, 459.3845 | notoginsenoside Fc |
| **62** | 18.87 | 1193.5969[b/f] | $C_{57}H_{94}O_{26}$ | 0.72 | 355.53 | −H | 1107.5960, 945.5431, 783.4903, 621.4369, 459.3849 | PPD-mal-4glc |
| **63** | 18.93 | 855.4754[b/e/f] | $C_{44}H_{72}O_{16}$ | 0.75 | 302.22 | −H | 769.4748, 475.3794 | PPT-mal-xyl-glc |
| **64** | 19.43 | 1123.5893[a] | $C_{53}H_{90}O_{22}$ | 0.37 | 275.15 | +HCOO | 1077.5830, 945.5417, 783.4889, 653.4628, 537.3445, 459.3856 | ginsenoside Rc |
| **65** | 19.80 | 1193.5956[b/f] | $C_{57}H_{94}O_{26}$ | −0.38 | 214.50 | −H | 1149.6067, 1107.5959, 945.5434, 783.4903, 621.4369, 459.3848 | PPD-mal-4glc |
| **66** | 19.86 | 1255.6317[a] | $C_{58}H_{98}O_{26}$ | 0.32 | 363.00 | +HCOO | 1209.6260, 1077.5844, 945.5436, 783.4892, 621.4370, 459.3845 | ginsenoside Ra1 |
| **67** | 20.19 | 1295.6268[b/e/f] | $C_{61}H_{100}O_{29}$ | −0.73 | 366.26 | −H | 1209.6269, 1077.5858, 945.5431, 783.4894, 621.4375, 459.3847 | PPD-mal-2xyl-3glc |
| **68** | 20.25 | 1193.5952[b/e/f] | $C_{57}H_{94}O_{26}$ | −0.68 | – | −H | 1149.6070, 1107.5955, 945.5430, 783.4901, 621.4373, 459.3847 | PPD-mal-4glc |

| | | | | | | | | |
|---|---|---|---|---|---|---|---|---|
| **69** | 20.43 | 1193.5946[c/e/f] | $C_{57}H_{94}O_{26}$ | −1.22 | 350.45 | −H | 1149.6064, 1107.5957, 945.5432, 783.4893, 621.4378, 459.3828 | PPD-mal-4glc |
| **70** | 20.67 | 1193.5948[b/e/f] | $C_{57}H_{94}O_{26}$ | −1.07 | – | −H | 1107.5958, 945.5429, 783.4896, 621.4370, 459.3839 | PPD-mal-4glc |
| **71** | 21.11 | 1387.6721[a] | $C_{63}H_{106}O_{30}$ | −1.5 | 381.91 | +HCOO | 1341.6676, 1209.6257, 1077.5843, 945.5428, 783.4897, 621.4367, 459.3850 | notoginsenoside S |
| **72** | 21.42 | 1163.5851[b/e/f] | $C_{56}H_{92}O_{25}$ | −0.34 | – | −H | 1077.5927, 945.5433, 783.4911, 621.4375, 459.3808 | PPD-mal-xyl-3glc |
| **73** | 21.55 | 683.4455[a] | $C_{36}H_{62}O_{9}$ | −0.48 | 275.30 | +HCOO | 637.4327, 621.4337, 475.3801, 391.2873 | ginsenoside F1 |
| **74** | 21.68 | 1163.5860[a] | $C_{56}H_{92}O_{25}$ | 0.44 | 348.79 | −H | 1077.5850, 1059.5755, 945.5441, 783.4900, 621.4362, 459.3845 | malonyl-ginsenoside Rc |
| **75** | 21.77 | 1295.6272[b/e/f] | $C_{61}H_{100}O_{29}$ | −0.42 | 374.67 | −H | 1209.6284, 1077.5854, 945.5435, 783.4897, 621.4372, 459.3849 | PPD-mal-2xyl-3glc |
| **76** | 21.85 | 1195.6267[d/f] | $C_{56}H_{94}O_{24}$ | −0.28 | 370.43 | +HCOO | 1149.6059, 1107.5978, 945.5462, 783.4859, 621.4365, 459.3841 | quinquenoside R1 or isomer |
| **77** | 21.97 | 1163.5860[b/f] | $C_{56}H_{92}O_{25}$ | 0.44 | – | −H | 1077.5853, 945.5437, 783.4878, 621.4337, 459.3830 | PPD-mal-xyl-3glc |
| **78** | 22.04 | 1165.6157[b/f] | $C_{55}H_{92}O_{23}$ | −3.54 | 359.82 | +HCOO | 1119.5917, 1077.5852, 945.5415, 783.4886, 621.4348, 459.3859 | PPD-Ace-xyl-3glc |
| **79** | 22.07 | 1279.6151[b/e/f] | $C_{57}H_{100}O_{31}$ | −1.94 | 282.37 | −H | 1107.5958, 945.5442, 783.4866, 621.4349, 459.3873 | PPD-Dimal-4glc |
| **80** | 22.09 | 1295.6435[b/e/f] | $C_{58}H_{104}O_{31}$ | −4.15 | 370.92 | −H | 1209.6264, 1077.5845, 945.5424, 783.4903, 621.4374, 459.3841 | PPD-mal-2xyl-3glc |
| **81** | 22.21 | 1163.5852[b/f] | $C_{56}H_{92}O_{25}$ | −0.22 | 342.77 | −H | 1077.5854, 945.5439, 783.4902, 621.4365, 459.3849 | PPD-mal-xyl-3glc |
| **82** | 22.23 | 1279.6135[b/e/f] | $C_{57}H_{100}O_{31}$ | −3.19 | 282.37 | −H | 1107.5959, 945.5433, 783.4897, 621.4363, 459.3831 | PPD-Dimal-4glc |
| **83** | 22.24 | 1119.5961[c/f] | $C_{55}H_{92}O_{23}$ | 0.39 | 353.83 | −H | 1077.5854, 783.4904, 621.4381, 459.3856 | PPD-Ace-xyl-3glc |
| **84** | 22.59 | 1123.5885[a] | $C_{53}H_{90}O_{22}$ | 0.04 | 359.67 | +HCOO | 1077.5821, 945.5406, 783.4878, 621.4378, 459.3845 | ginsenoside Rb2 |
| **85** | 22.86 | 1295.6253[b/e/f] | $C_{61}H_{100}O_{29}$ | −1.89 | 362.70 | −H | 1209.6273, 1077.5848, 945.5426, 783.4900, 621.4372, 459.3836 | PPD-mal-2xyl-3glc |
| **86** | 22.87 | 1119.5961[c/e/f] | $C_{55}H_{92}O_{23}$ | 0.39 | 353.17 | −H | 1077.5853, 945.5441, 783.4903, 621.4367, 459.3849 | PPD-Ace-xyl-3glc |
| **87** | 23.04 | 1163.5846[b/e/f] | $C_{56}H_{92}O_{25}$ | −0.77 | – | −H | 1077.5843, 945.5442, 783.4853, 621.4353, 459.3890 | PPD-mal-xyl-3glc |
| **88** | 23.31 | 1163.5840[b/e/f] | $C_{56}H_{92}O_{25}$ | −1.31 | 351.16 | −H | 1077.5836, 945.5427, 783.4899, 621.4361, 459.3848 | PPD-mal-xyl-3glc |
| **89** | 23.66 | 1295.6442[b/e/f] | $C_{58}H_{104}O_{31}$ | −3.61 | 369.48 | −H | 1209.6276, 1077.5843, 945.5437, 783.4896, 621.4360, 459.3852 | PPD-mal-2xyl-3glc |
| **90** | 23.69 | 1123.5891[a] | $C_{53}H_{90}O_{22}$ | −1.29 | 347.37 | +HCOO | 1077.5834, 945.5422, 783.4891, 621.4364, 459.3842 | ginsenoside Rb3 |
| **91** | 23.93 | 1279.5942[b/e/f] | $C_{60}H_{96}O_{29}$ | −1.76 | 278.92 | −H | 1107.5952, 945.5413, 783.4886, 621.4369, 459.3841 | PPD-Dimal-4glc |
| **92** | 23.97 | 1123.5891[d/f] | $C_{53}H_{90}O_{22}$ | −1.36 | 357.13 | +HCOO | 1077.5840, 945.5433, 783.4892, 621.4366, 459.3841 | ginsenoside Rc or isomer |
| **93** | 23.99 | 1295.6437[b/e/f] | $C_{58}H_{104}O_{31}$ | −4.00 | 374.68 | −H | 1209.6282, 1077.5855, 945.5428, 783.4889, 621.4371, 459.3855 | PPD-mal-2xyl-3glc |
| **94** | 24.27 | 1295.6269[b/e/f] | $C_{61}H_{100}O_{29}$ | −0.66 | – | −H | 1209.6274, 1077.5854, 945.5438, 783.4898, 621.4371, 459.3848 | PPD-mal-2xyl-3glc |

| | | | | | | | | |
|---|---|---|---|---|---|---|---|---|
| **95** | 24.64 | 1295.6260[b/e/f] | $C_{61}H_{100}O_{29}$ | −1.35 | 365.66 | −H | 1209.6266, 1077.5850, 945.5430, 783.4895, 621.4371, 459.3844 | PPD-mal-2xyl-3glc |
| **96** | 24.67 | 1163.5858[a] | $C_{56}H_{92}O_{25}$ | 0.26 | 354.92 | −H | 1077.5860, 1059.5721, 945.5433, 783.4896, 621.4357, 459.3852 | malonylginsenoside Rb2 |
| **97** | 24.81 | 1123.6076[d/f] | $C_{53}H_{90}O_{22}$ | 1.30 | 357.12 | +HCOO | 1077.5865, 945.5421, 783.4893, 621.4356, 459.3807 | notoginsenoside L or isomer |
| **98** | 24.85 | 1255.6309[d/f] | $C_{58}H_{98}O_{26}$ | −1.45 | 284.63 | +HCOO | 1209.6256, 1077.5844, 945.5424, 783.4896, 621.4362, 459.3839 | notoginsenoside-FZ or isomer |
| **99** | 25.41 | 1165.5999[b/e/f] | $C_{55}H_{92}O_{23}$ | −2.64 | 364.03 | +HCOO | 1119.5927, 1077.5849, 945.5417, 783.4885, 621.4369, 459.3845 | PPD-Ace-xyl-3glc |
| **100** | 25.43 | 1137.6066[d/f] | $C_{54}H_{92}O_{22}$ | 2.06 | 349.98 | +HCOO | 1091.6030, 945.5491, 783.4910, 621.4379, 459.3836 | gypenoside V or isomer |
| **101** | 25.63 | 1249.5844[b/e/f] | $C_{59}H_{94}O_{28}$ | −1.19 | 358.25 | −H | 1077.5835, 945.5442, 783.4898, 621.4364, 459.3829 | PPD-Dimal-xyl-3glc |
| **102** | 25.68 | 1163.5844[b/e/f] | $C_{56}H_{92}O_{25}$ | −0.90 | 346.28 | −H | 1077.5844, 945.5423, 783.4899, 621.4364, 459.3845 | PPD-mal-xyl-3glc |
| **103** | 25.83 | 1119.5956[c/e/f] | $C_{55}H_{92}O_{23}$ | −0.05 | 278.92 | −H | 1077.5846, 945.5440, 783.4895, 621.4361, 459.3856 | PPD-Ace-xyl-3glc |
| **104** | 25.95 | 1163.5842[b/e/f] | $C_{56}H_{92}O_{25}$ | −1.11 | 279.06 | −H | 1077.5843, 945.5430, 783.4901, 621.4371, 459.3841 | PPD-mal-xyl-3glc |
| **105** | 26.03 | 1205.5949[b/e/f] | $C_{58}H_{94}O_{26}$ | −0.96 | 347.64 | −H | 1119.5936, 1077.5877, 945.5422, 783.4886, 621.4379, 459.3833 | PPD-mal-Ace-xyl-3glc |
| **106** | 26.24 | 1163.5833[b/e/f] | $C_{56}H_{92}O_{25}$ | −1.92 | 343.52 | −H | 1077.5840, 945.5425, 783.4895, 621.4366, 459.3842 | PPD-mal-xyl-3glc |
| **107** | 26.97 | 1119.5969[c/f] | $C_{55}H_{92}O_{23}$ | 1.11 | 365.10 | −H | 1077.5856, 945.5439, 783.4910, 459.3843 | PPD-Ace-xyl-3glc |
| **108** | 27.13 | 1163.5837[b/e/f] | $C_{56}H_{92}O_{25}$ | −1.50 | 267.37 | −H | 1077.5833, 945.5418, 783.4889, 621.4364, 459.3841 | PPD-mal-xyl-3glc |
| **109** | 27.34 | 1119.5975[c/f] | $C_{55}H_{92}O_{23}$ | 1.64 | 334.07 | −H | 1077.5852, 945.5430, 783.4901, 621.4375, 459.3837 | PPD-Ace-xyl-3glc |
| **110** | 27.35 | 1163.5827[c/e/f] | $C_{56}H_{92}O_{25}$ | −2.40 | 342.82 | −H | 1077.5852, 945.5430, 783.4901, 621.4375, 459.3837 | PPD-mal-xyl-3glc |
| **111** | 27.67 | 1163.5836[b/e/f] | $C_{56}H_{92}O_{25}$ | −1.66 | 349.64 | −H | 1077.5842, 945.5424, 783.4889, 621.4363, 459.3842 | PPD-mal-xyl-3glc |
| **112** | 27.75 | 1249.5840[c/e/f] | $C_{59}H_{94}O_{28}$ | −1.51 | 358.93 | −H | 1077.5847, 945.5433, 621.4371, 459.3852 | PPD-Dimal-xyl-3glc |
| **113** | 27.95 | 1163.5853[b/f] | $C_{56}H_{92}O_{25}$ | −0.16 | 278.78 | −H | 1077.5831, 945.5420, 783.4892, 621.4264, 459.3834 | PPD-mal-xyl-3glc |
| **114** | 28.24 | 1163.5837[b/e/f] | $C_{56}H_{92}O_{25}$ | −1.53 | 287.08 | −H | 1077.5835, 945.5424, 783.4891, 621.4364, 459.3843 | PPD-mal-xyl-3glc |
| **115** | 28.40 | 1295.6251[b/e/f] | $C_{61}H_{100}O_{29}$ | −2.04 | 359.27 | −H | 1209.6257, 1077.5825, 945.5415, 783.4886, 621.4358, 459.3844 | PPD-mal-2xyl-3glc |
| **116** | 28.71 | 991.5467[a] | $C_{48}H_{82}O_{18}$ | −1.20 | 331.74 | +HCOO | 945.5414, 783.4889, 621.4363, 459.3844 | ginsenoside Rd |
| **117** | 28.94 | 1163.5860[b/f] | $C_{56}H_{92}O_{25}$ | 0.44 | 362.64 | −H | 1077.5866, 945.5359, 783.4867, 621.4315, 459.3817 | PPD-mal-xyl-3glc |
| **118** | 29.19 | 1249.5853[b/e/f] | $C_{59}H_{94}O_{28}$ | −0.47 | 356.61 | −H | 1077.5860, 945.5427, 783.4894, 621.4360, 459.3845 | PPD-Dimal-xyl-3glc |
| **119** | 29.49 | 1205.5971[b/e/f] | $C_{58}H_{94}O_{26}$ | 0.86 | 346.54 | −H | 1119.5960, 1077.5864, 945.5445, 783.4911, 621.4378, 459.3840 | PPD-mal-Ace-xyl-3glc |
| **120** | 29.76 | 1093.5802[b/e/f] | $C_{53}H_{90}O_{23}$ | 0.17 | 351.04 | −H | 1047.5738, 915.5288, 753.4805, 621.4349, 459.3830 | PPD-2xyl-2glc |

| | | | | | | | | |
|---|---|---|---|---|---|---|---|---|
| **121** | 29.95 | 1249.5857[b/e/f] | $C_{59}H_{94}O_{28}$ | –0.15 | 359.08 | –H | 1077.5859, 945.5401, 783.4889, 621.4357, 459.3859 | PPD-Dimal-xyl-3glc |
| **122** | 29.99 | 1205.5949[b/e/f] | $C_{58}H_{94}O_{26}$ | –0.96 | 368.19 | –H | 1119.5939, 1077.5868, 945.5465, 783.4856, 621.4373, 459.3851 | PPD-mal-Ace-xyl-3glc |
| **123** | 30.26 | 1249.5862[b/e/f] | $C_{59}H_{94}O_{28}$ | 0.25 | – | –H | 1077.5860, 945.5461, 783.4899, 621.4377, 459.3850 | PPD-Dimal-xyl-3glc |
| **124** | 30.40 | 1205.5978[b/e/f] | $C_{58}H_{94}O_{26}$ | 1.44 | 357 .28 | –H | 1119.5965, 1077.5849, 945.5417, 783.4902, 621.4393, 459.3855 | PPD-mal-Ace-xyl-3glc |
| **125** | 30.51 | 1207.6108[b/e/f] | $C_{57}H_{94}O_{24}$ | 2.04 | 389.96 | +HCOO | 1161.6086, 1119.5955, 1077.5862, 783.4927, 621.4374, 459.3842 | PPD-2Ace-xyl-3glc |
| **126** | 30.63 | 1249.5826[b/e/f] | $C_{59}H_{94}O_{28}$ | –2.63 | 363.75 | –H | 1077.5864, 945.5442, 783.4905, 621.4372, 459.3839 | PPD-Dimal-xyl-3glc |
| **127** | 30.81 | 1031.5443[a] | $C_{51}H_{84}O_{21}$ | 1.00 | 340.41 | –H | 945.5435, 783.4904, 621.4387, 459.3845 | malonylginsenoside Rd |
| **128** | 30.91 | 1093.5811[b/e/f] | $C_{53}H_{90}O_{23}$ | 1.00 | 347.43 | –H | 1047.5737, 915.5361, 753.4784, 621.4368, 459.3859 | PPD-2xyl-2glc |
| **129** | 30.98 | 1249.5873[b/e/f] | $C_{59}H_{94}O_{28}$ | 1.13 | – | –H | 1077.5864, 945.5387, 783.4928, 621.4345, 459.3815 | PPD-Dimal-xyl-3glc |
| **130** | 31.04 | 1093.5818[b/f] | $C_{52}H_{88}O_{21}$ | –1.75 | 347.43 | +HCOO | 1047.5727, 915.5441, 753.4770, 621.4371, 459.3876 | notoginsenoside O or isomer |
| **131** | 31.10 | 1205.5955[b/e/f] | $C_{58}H_{94}O_{26}$ | –0.46 | 351.34 | –H | 1119.5958, 1077.5868, 945.5441, 783.4876, 621.4343, 459.3878 | PPD-mal-Ace-xyl-3glc |
| **132** | 31.25 | 1249.5855[b/e/f] | $C_{59}H_{94}O_{28}$ | –0.31 | 363.76 | –H | 1077.5858, 945.5440, 783.4906, 621.4361, 459.3842 | PPD-Dimal-xyl-3glc |
| **133** | 31.35 | 1205.5974[b/e/f] | $C_{58}H_{94}O_{26}$ | 1.11 | 351.34 | –H | 1119.5970, 1077.5855, 945.5436, 783.4907, 621.4373, 459.3851 | PPD-mal-Ace-xyl-3glc |
| **134** | 31.55 | 1165.6015[b/e/f] | $C_{55}H_{92}O_{23}$ | –1.22 | 363.62 | +HCOO | 1119.5943, 1077.5847, 945.5485, 783.4889, 621.4335, 459.3856 | PPD-Ace-xyl-3glc |
| **135** | 31.66 | 1249.5846[b/e/f] | $C_{59}H_{94}O_{28}$ | –1.03 | 357.75 | –H | 1077.5854, 945.5431, 783.4903, 621.4370, 459.3844 | PPD-Dimal-xyl-3glc |
| **136** | 31.67 | 1205.5964[b/e/f] | $C_{58}H_{94}O_{26}$ | 0.28 | 346.77 | –H | 1119.5955, 1077.5848, 945.5429, 783.4906, 621.4368, 459.3847 | PPD-mal-Ace-xyl-3glc |
| **137** | 31.90 | 1031.5420[d/f] | $C_{51}H_{84}O_{21}$ | –1.20 | 323.41 | –H | 945.5417, 783.4897, 621.4367, 459.3845 | malonylfloralginsenoside Rd4 or isomer |
| **138** | 31.94 | 1121.4425[c/e/f] | $C_{43}H_{78}O_{33}$ | 6.46 | 330.93 | –H | 945.5424, 783.4906, 459.3844 | PPD-glurA-3glc |
| **139** | 31.94 | 991.5484[c/f] | $C_{48}H_{82}O_{18}$ | –0.46 | 332.37 | +HCOO | 945.5424, 783.4906, 621.4371, 459.3844 | chikusetsusaponin FK7 or isomer |
| **140** | 32.17 | 1033.5569[b/e/f] | $C_{50}H_{84}O_{19}$ | 1.01 | 213.18 | +HCOO | 987.5544, 945.5428, 783.4905, 621.4370, 459.3838 | PPD-Ace-3glc |
| **141** | 32.44 | 1265.6203[b/e/f] | $C_{60}H_{98}O_{28}$ | 2.46 | 362.88 | –H | 1179.6118, 1047.5701, 915.5317, 783.4894, 621.4398, 459.3867 | PPD-mal-3xyl-2glc |
| **142** | 32.55 | 987.5539[c/f] | $C_{50}H_{84}O_{19}$ | 0.50 | 340.81 | –H | 945.5423, 783.4894, 621.4376, 459.3848 | PPD-Ace-3glc |
| **143** | 32.65 | 1205.5984[b/e/f] | $C_{58}H_{94}O_{26}$ | 1.94 | 349.93 | –H | 1119.6081, 1077.5871, 945.5346, 783.4859, 621.4466, 459.3820 | PPD-mal-Ace-xyl-3glc |
| **144** | 32.92 | 769.4389[d/f] | $C_{40}H_{66}O_{14}$ | 1.20 | 284.23 | –H | 769.4389, 637.4305, 475.3798, 391.2861 | 20(*S*)-sanchirhinoside A3 or isomer |
| **145** | 33.07 | 1165.6027[b/e/f] | $C_{55}H_{92}O_{23}$ | 0.21 | 372.42 | +HCOO | 1119.5959, 1077.5866, 945.5435, 783.4903, 621.4378, 459.3818 | PPD-Ace-xyl-3glc |

| | | | | | | | | |
|---|---|---|---|---|---|---|---|---|
| **146** | 33.16 | 1205.5956[b/e/f] | $C_{58}H_{94}O_{26}$ | –0.38 | 360.77 | –H | 1119.6002, 1077.5904, 945.5433, 783.4878, 621.4329, 459.3829 | PPD-mal-Ace-xyl-3glc |
| **147** | 33.25 | 1033.5594[c/e/f] | $C_{50}H_{84}O_{19}$ | –0.41 | 344.24 | +HCOO | 987.5530, 945.5432, 783.4897, 621.4373, 459.3846 | PPD-Ace-3glc |
| **148** | 33.27 | 1073.5543[b/e/f] | $C_{53}H_{86}O_{22}$ | 0.47 | 352.29 | –H | 987.5548, 945.5431, 783.4909, 621.4364, 459.3853 | PPD-mal-Ace-3glc |
| **149** | 33.31 | 1117.5441[b/e/f] | $C_{54}H_{86}O_{24}$ | 0.44 | 337.86 | –H | 945.5423, 783.4903, 621.4373, 459.3848 | PPD-Dimal-3glc |
| **150** | 33.36 | 1075.5705[b/e/f] | $C_{52}H_{86}O_{20}$ | 0.94 | 353.87 | +HCOO | 1029.5621, 987.5532, 945.5446, 783.4933, 621.4358, 459.3846 | PPD-2Ace-3glc |
| **151** | 33.42 | 1093.5821[d/f] | $C_{52}H_{88}O_{21}$ | 1.95 | 338.31 | +HCOO | 1047.5744, 915.5307, 783.4891, 621.4413, 459.3842 | notoginsenoside P or isomer |
| **152** | 33.57 | 991.5488[a] | $C_{48}H_{82}O_{18}$ | –0.15 | 321.69 | +HCOO | 945.5427, 927.5316, 783.4927, 621.4397, 459.3835 | gypenoside XVⅡ |
| **153** | 33.59 | 1117.5444[b/e/f] | $C_{54}H_{86}O_{24}$ | 0.66 | 342.21 | –H | 945.5448, 621.4384, 459.3850 | PPD-Dimal-3glc |
| **154** | 33.62 | 1033.5746[b/f] | $C_{50}H_{84}O_{19}$ | 0.50 | 341.25 | +HCOO | 987.5539, 945.5434, 783.4908, 621.4373, 459.3844 | PPD-Ace-3glc |
| **155** | 33.64 | 1031.5442[d/f] | $C_{51}H_{84}O_{21}$ | 0.90 | 331.98 | –H | 945.5425, 783.4914, 621.4372, 459.3853 | malonylfloralginsenoside Rd3 or isomer |
| **156** | 33.68 | 1047.5745[c/f] | $C_{52}H_{88}O_{21}$ | –0.03 | 348.56 | –H | 915.5324, 783.4896, 621.4372, 459.3845 | notoginsenoside P or isomer |
| **157** | 33.73 | 1133.5780[b/e/f] | $C_{55}H_{90}O_{24}$ | 2.71 | 339.22 | –H | 1047.5774, 915.5325, 783.4870, 621.4406, 459.3867 | PPD-mal-2xyl-2glc |
| **158** | 33.74 | 1093.5808[b/e/f] | $C_{53}H_{90}O_{23}$ | 0.68 | 350.95 | –H | 1047.5753, 915.5323, 783.4909, 621.4373, 459.3840 | PPD-2xyl-2glc |
| **159** | 33.76 | 1165.6014[b/e/f] | $C_{55}H_{92}O_{23}$ | 1.91 | 372.70 | +HCOO | 1119.5978, 1077.5856, 945.5436, 783.4907, 621.4372, 459.3863 | PPD-Ace-xyl-3glc |
| **160** | 33.87 | 1075.5682[b/e/f] | $C_{52}H_{86}O_{20}$ | –1.17 | 359.94 | +HCOO | 1029.5632, 987.5533, 945.5435, 621.4370, 459.3854 | PPD-2Ace-3glc |
| **161** | 33.94 | 1117.5432[b/e/f] | $C_{54}H_{86}O_{24}$ | –0.39 | 337.74 | –H | 945.5437, 783.4904, 621.4372, 459.3847 | PPD-Dimal-3glc |
| **162** | 33.94 | 1073.5715[b/e/f] | $C_{57}H_{86}O_{19}$ | 2.28 | 207.00 | –H | 987.5531, 945.5425, 783.4890, 621.4369, 459.3849 | PPD-mal-Ace-3glc |
| **163** | 34.06 | 1075.5842[b/e/f] | $C_{52}H_{86}O_{20}$ | 5.86 | 359.94 | +HCOO | 1029.5700, 987.5529, 945.5418, 783.4983, 621.4390, 459.3864 | PPD-2Ace-3glc |
| **164** | 34.08 | 1031.5460[d/f] | $C_{51}H_{84}O_{21}$ | 2.68 | 338.12 | –H | 945.5442, 783.4894, 621.4401, 459.3868 | malonylfloralginsenoside Rd1 or isomer |
| **165** | 34.14 | 961.5397[d/f] | $C_{47}H_{80}O_{17}$ | 2.00 | 325.41 | +HCOO | 915.5298, 783.4868, 621.4379, 459.3836 | vinaginsenoside R17 or isomer |
| **166** | 34.17 | 1117.5445[b/e/f] | $C_{54}H_{86}O_{24}$ | 0.81 | 338.33 | –H | 945.5438, 765.4731, 621.4294, 459.3874 | PPD-Dimal-3glc |
| **167** | 34.18 | 1073.5557[b/e/f] | $C_{53}H_{86}O_{22}$ | 1.77 | 351.78 | –H | 987.5534, 945.5453, 783.4893, 621.4348, 459.3862 | PPD-mal-Ace-3glc |
| **168** | 34.23 | 1093.5818[b/e/f] | $C_{53}H_{90}O_{23}$ | 1.61 | 337.32 | –H | 1047.5712, 915.5339, 783.5080, 621.4369, 459.3815 | PPD-2xyl-2glc |
| **169** | 34.26 | 1205.5983[b/e/f] | $C_{58}H_{94}O_{26}$ | 1.86 | 349.05 | –H | 1119.5945, 1077.5852, 915.5349, 783.4860, 621.4381, 459.3853 | PPD-mal-Ace-3glc-xyl |
| **170** | 34.47 | 1115.5664[b/e/f] | $C_{55}H_{88}O_{23}$ | 1.83 | 362.71 | –H | 1029.5652, 987.5533, 945.5428, 783.4873, 621.4390, 459.3847 | PPD-mal-2Ace-3glc |
| **171** | 34.47 | 1159.5549[c/e/f] | $C_{56}H_{88}O_{25}$ | 0.61 | 352.77 | –H | 987.5538, 945.5429, 783.4900, 621.4362, 459.3837 | PPD-Dimal-Ace-3glc |

| | | | | | | | | |
|---|---|---|---|---|---|---|---|---|
| **172** | 34.48 | 1119.5673[c/e/f] | $C_{58}H_{88}O_{21}$ | –6.46 | 351.45 | –H | 987.5538, 945.5429, 783.4900, 621.4362, 459.3837 | PPD-xyl-Ace-3glc |
| **173** | 34.50 | 1117.5581[b/f] | $C_{51}H_{86}O_{21}$ | –0.70 | – | –H | 945.5441, 783.4869, 621.4386, 459.3821 | PPD-Dimal-3glc |
| **174** | 34.56 | 1075.5715[b/e/f] | $C_{52}H_{86}O_{20}$ | 1.94 | 350.72 | +HCOO | 1029.5595, 987.5542, 945.5452, 783.4876, 621.4364, 459.3855 | PPD-2Ace-3glc |
| **175** | 34.62 | 1205.5963[b/e/f] | $C_{58}H_{94}O_{26}$ | 0.20 | 348.95 | –H | 1119.5990, 1077.5862, 945.5453, 783.4897, 621.4371, 459.3859 | PPD-mal-Ace-xyl-3glc |
| **176** | 34.65 | 1031.5446[d/f] | $C_{51}H_{84}O_{21}$ | 1.33 | 328.62 | –H | 945.5423, 783.489, 621.4371, 459.3829 | malonylfloralginsenoside Rd2 or isomer |
| **177** | 34.72 | 1117.5443[b/e/f] | $C_{54}H_{86}O_{24}$ | 0.60 | 354.91 | –H | 945.5452, 783.4901, 621.4369, 459.3809 | PPD-Dimal-3glc |
| **178** | 34.73 | 1073.5553[b/e/f] | $C_{53}H_{86}O_{22}$ | 1.40 | 354.93 | –H | 987.5546, 945.5414, 783.4906, 621.4337, 459.3851 | PPD-mal-Ace-3glc |
| **179** | 34.79 | 961.5373[a] | $C_{47}H_{80}O_{17}$ | –2.92 | 322.31 | +HCOO | 915.5296, 783.4889, 621.4361, 459.3817 | notoginsenoside Fe |
| **180** | 34.84 | 1133.5785[b/e/f] | $C_{55}H_{90}O_{24}$ | 3.15 | 340.64 | –H | 957.5421, 915.5311, 783.4892, 621.4408, 459.3806 | PPD-glurA-Ace-xyl-2glc |
| **181** | 34.86 | 961.5378[a] | $C_{47}H_{80}O_{17}$ | –0.11 | 325.83 | +HCOO | 915.5301, 783.4895, 621.4356, 459.3857 | notoginsenoside Fd |
| **182** | 34.87 | 1131.4683[b/e/f] | $C_{49}H_{80}O_{29}$ | –2.61 | 334.97 | –H | 1089.5933, 957.5457, 915.5326, 783.4870, 621.4336, 459.3895 | PPD-2Ace-2xyl-2glc |
| **183** | 35.13 | 1133.5763[b/e/f] | $C_{55}H_{90}O_{24}$ | 1.21 | 334.2 | –H | 1047.5714, 915.5313, 783.4956, 621.4364, 459.3836 | PPD-mal-2xyl-2glc |
| **184** | 35.14 | 957.5456[c/e/f] | $C_{49}H_{82}O_{18}$ | 2.88 | 332.53 | –H | 915.5307, 783.4903, 621.4365, 459.3840 | PPD-xyl-2glc |
| **185** | 35.29 | 961.5381[a] | $C_{47}H_{80}O_{17}$ | 0.32 | 322.73 | +HCOO | 915.5321, 762.4102, 621.4382, 459.3866 | ginsenoside Rd2 |
| **186** | 35.43 | 1001.5333[b/e/f] | $C_{50}H_{82}O_{20}$ | 0.63 | 319.34 | –H | 915.5328, 783.4955, 621.4380, 459.3850 | PPD-mal-xyl-2glc |
| **187** | 35.54 | 1117.5466[b/e/f] | $C_{54}H_{86}O_{24}$ | 2.66 | 332.19 | –H | 945.5385, 783.4889, 621.4386, 459.3772 | PPD-Dimal-3glc |
| **188** | 35.56 | 1107.5993[d/f] | $C_{54}H_{92}O_{23}$ | 3.30 | 350.34 | –H | 1061.5912, 929.5502, 783.4905, 621.4363, 459.3829 | ginsenoside Rb4 or isomer |
| **189** | 35.60 | 1093.5801[b/e/f] | $C_{53}H_{90}O_{23}$ | 0.12 | 338.01 | –H | 1047.5758, 915.5321, 783.4890, 621.4381, 459.3849 | PPD-2xyl-2glc |
| **190** | 35.62 | 1003.5460[c/e/f] | $C_{49}H_{82}O_{18}$ | 0.06 | 339.24 | +HCOO | 957.5429, 825.5001, 783.4907, 621.4374, 459.3841 | PPD-xyl-Ace-2glc |
| **191** | 35.62 | 957.5430[c/e/f] | $C_{49}H_{82}O_{18}$ | 0.17 | 333.21 | –H | 915.5324, 783.4907, 621.4374, 459.3841 | PPD-Ace-xyl-2glc |
| **192** | 35.83 | 1033.5607[b/f] | $C_{50}H_{84}O_{19}$ | 0.81 | 344.07 | +HCOO | 987.5542, 945.5422, 783.4919, 621.4376, 459.3827 | PPD-Ace-3glc |
| **193** | 35.91 | 1003.5460[c/e/f] | $C_{49}H_{82}O_{18}$ | 0.17 | 331.47 | +HCOO | 957.5430, 825.4987, 783.4922, 621.4386, 459.3854 | PPD-xyl-Ace-2glc |
| **194** | 35.94 | 1087.5342[b/e/f] | $C_{53}H_{84}O_{23}$ | 1.06 | 335.49 | –H | 915.5337, 783.4925, 621.4376, 459.3854 | PPD-Dimal--xyl-2glc |
| **195** | 36.03 | 1087.5500[b/e/f] | $C_{57}H_{84}O_{20}$ | 1.55 | 335.49 | –H | 915.5311, 783.5023, 621.4356, 459.3858 | PPD-Dimal-xyl-2glc |
| **196** | 36.14 | 1001.5326[b/e/f] | $C_{50}H_{82}O_{20}$ | -0.07 | 331.19 | –H | 915.5347, 783.4903, 621.4380, 459.3852 | PPD-mal-xyl-2glc |
| **197** | 36.27 | 1087.5342[b/e/f] | $C_{53}H_{84}O_{23}$ | 1.05 | 326.23 | –H | 915.5283, 783.4935, 621.4361, 459.3896 | PPD-Dimal-xyl-2glc |
| **198** | 36.40 | 1001.5322[b/e/f] | $C_{50}H_{82}O_{20}$ | –0.47 | 317.59 | –H | 915.5261, 783.4832, 621.4363, 459.3820 | PPD-xyl-2glc |
| **199** | 36.43 | 975.5680[b/e/f] | $C_{48}H_{82}O_{17}$ | 2.77 | 329.90 | +HCOO | 929.5505, 783.4911, 621.4366, 459.3833 | PPD-rha-2glc |
| **200** | 36.51 | 1073.5566[b/e/f] | $C_{53}H_{86}O_{22}$ | 2.61 | 354.99 | –H | 987.5517, 945.5475, 783.4876, 621.4338, 459.3860 | PPD-mal-Ace-3glc |
| **201** | 36.74 | 1087.5351[b/e/f] | $C_{53}H_{84}O_{23}$ | 1.85 | 329.07 | –H | 915.5280, 783.4866, 621.4361, 459.3827 | PPD-Dimal-xyl-2glc |

| | | | | | | | | |
|---|---|---|---|---|---|---|---|---|
| **202** | 36.84 | 829.4961[a] | $C_{42}H_{72}O_{13}$ | 0.73 | 306.82 | +HCOO | 783.4929, 621.4385, 459.3844, 375.2900 | ginsenoside F2 |
| **203** | 37.07 | 1015.5495[b/e/f] | $C_{51}H_{84}O_{20}$ | 1.16 | 339.42 | –H | 929.5497, 783.4898, 621.4363, 459.3850 | PPD-mal-rha-2glc |
| **204** | 37.15 | 1101.5507[b/e/f] | $C_{54}H_{86}O_{23}$ | 1.82 | 341.83 | –H | 929.5503, 783.4887, 621.4401, 459.3838 | PPD-Dimal--rha-2glc |
| **205** | 37.18 | 871.5053[b/e/f] | $C_{44}H_{74}O_{14}$ | 1.96 | 317.61 | +HCOO | 825.5022, 783.4867, 763.4077, 621.4384, 459.3845 | PPD-Ace-2glc |
| **206** | 37.28 | 1015.5488[b/e/f] | $C_{51}H_{84}O_{20}$ | 0.48 | 336.21 | –H | 929.5489, 783.4937, 621.4384, 459.3852 | PPD-mal-rha-2glc |
| **207** | 37.43 | 1059.5769[d/f] | $C_{52}H_{86}O_{19}$ | –0.94 | 352.06 | +HCOO | 1013.5681, 945.5435, 783.4935, 621.4352, 459.3854 | quinquenoside I or isomer |
| **208** | 37.67 | 869.4914[b/e/f] | $C_{45}H_{74}O_{18}$ | 1.14 | 310.02 | –H | 783.4901 621.4354, 459.3837 | PPD-mal-2glc |
| **209** | 37.75 | 871.5049[b/e/f] | $C_{44}H_{74}O_{14}$ | –3.13 | 318.66 | +HCOO | 825.4980, 783.4939, 621.4325, 459.3869 | PPD-Ace-2glc |
| **210** | 37.83 | 829.4954[a] | $C_{43}H_{74}O_{15}$ | –0.15 | 305.11 | +HCOO | 783.4895, 621.4386, 459.3843 | 20(*S*)-ginsenoside Rg3 |
| **211** | 37.93 | 955.4918[b/e/f] | $C_{48}H_{76}O_{19}$ | 1.03 | 314.77 | –H | 783.5115, 621.4362, 459.3832 | PPD-Dimal-2glc |
| **212** | 38.16 | 829.4950[a] | $C_{43}H_{74}O_{15}$ | –0.64 | 301.13 | +HCOO | 783.4913, 621.4358, 459.3842 | 20(*R*)-ginsenoside Rg3 |
| **213** | 38.44 | 871.5056[b/f] | $C_{44}H_{74}O_{14}$ | –0.95 | 320.28 | +HCOO | 825.4998, 783.4899, 621.4381, 459.3847 | PPD-Ace-2glc |
| **214** | 38.54 | 955.4925[b/e/f] | $C_{48}H_{76}O_{19}$ | 1.76 | 316.29 | –H | 783.4930, 621.4353, 459.3849 | PPD-Dimal-2glc |
| **215** | 38.58 | 869.4916[b/e/f] | $C_{45}H_{74}O_{18}$ | 1.37 | 306.02 | –H | 783.4867, 621.4305, 459.3837 | PPD-mal-2glc |
| **216** | 39.05 | 799.4858[b/e/f] | $C_{42}H_{72}O_{14}$ | 1.11 | 304.12 | –H | 753.4762, 621.4373, 459.3840 | PPD-xyl-glc |
| **217** | 39.45 | 799.4856[d/f] | $C_{42}H_{72}O_{14}$ | 0.88 | 304.95 | +HCOO | 753.4789, 621.4361, 459.3843 | gypenoside XIII or isomer |
| **218** | 40.76 | 811.4987[a] | $C_{42}H_{70}O_{12}$ | 1.76 | 315.78 | +HCOO | 765.4808, 603.4288 | ginsenoside Rk1 |
| **219** | 41.81 | 753.4446[d/f] | $C_{40}H_{66}O_{13}$ | 2.04 | 287.12 | –H | 621.4371, 459.3814 | gypenoside XIII or isomer |

a: Identification assisted with reference compounds comparison; b: Components identified in the "Unknown Components" section of the DDA data; c: Components identified in HDMS$^E$ data; d: Components identified in the "Identified Components" section of the DDA data; e: The reported components may not have been isolated from *Panax ginseng*; f: Tentative characterization by analyzing the high-accuracy MS$^2$ data.

**Table S3.** Information for the 40 prototype components characterized from rat plasma.

| No. | Observed RT (min) | Observed m/z | Formula | Mass error (ppm) | CCA (Å²) | Adducts | ESI-MS² | Identification |
|---|---|---|---|---|---|---|---|---|
| **4** | 5.25 | 977.5315[a] | $C_{47}H_{80}O_{18}$ | –1.15 | 322.41 | +HCOO | 931.5263, 799.4839, 637.4322, 475.3793, 391.2845 | notoginsenoside R1 |
| **6** | 5.65 | 991.5484[a] | $C_{48}H_{82}O_{18}$ | 0.06 | 327.00 | +HCOO | 945.5429, 799.4842, 637.4319, 619.4202, 475.3887, 391.2848 | ginsenoside Re |
| **7** | 5.69 | 845.4997[a] | $C_{42}H_{72}O_{14}$ | 0.21 | 297.89 | +HCOO | 799.4851, 637.4321, 475.3798, 391.2867 | ginsenoside Rg1 |
| **31** | 10.81 | 1285.6450[a] | $C_{59}H_{100}O_{27}$ | 0.95 | 372.56 | +HCOO | 1239.6391, 1107.5986, 945.5443, 783.4914, 621.4364, 459.3857, 353.1086 | notoginsenosides R4 |
| **38** | 12.84 | 1285.6410[a] | $C_{59}H_{100}O_{27}$ | –0.53 | 372.92 | +HCOO | 1239.6357, 1107.5938, 945.5416, 783.4887, 621.4366, 459.3876 | notoginsenoside Fa |
| **39** | 13.03 | 1341.6670[b] | $C_{63}H_{106}O_{30}$ | –2.32 | 384.79 | –H | 1341.6664, 1209.6253, 1077.5841, 945.5425, 783.4892, 621.4363, 459.3841 | PPD-3xyl-2glc |
| **48** | 15.40 | 1255.6320[a] | $C_{58}H_{98}O_{26}$ | 0.67 | 365.25 | +HCOO | 1209.6261, 1077.5841, 945.5425, 783.4896, 621.4359, 459.3872 | notoginsenoside FP2 |
| **49** | 15.63 | 1387.6910[a] | $C_{63}H_{106}O_{30}$ | –0.53 | 302.07 | +HCOO | 1341.6689, 1209.6272, 1077.5856, 945.5442, 783.4902, 765.4810, 621.4379, 459.3851 | notoginsenoside Fh1 |
| **50** | 16.10 | 1153.6020[a] | $C_{54}H_{92}O_{23}$ | –0.60 | 355.55 | +HCOO | 1107.5950, 1089.5862, 945.5424, 783.4899, 621.4378, 459.3842 | ginsenoside Rb1 |
| **51** | 16.26 | 1325.6380[b] | $C_{62}H_{102}O_{30}$ | –0.58 | 373.85 | –H | 1239.6383, 1107.5957, 945.5436, 783.4907, 621.4373, 459.3849 | malonylginsenoside Ra3 or isomer |
| **52** | 16.38 | 1255.6320[b] | $C_{58}H_{98}O_{26}$ | 0.12 | 361.25 | +HCOO | 1209.6275, 1077.5861, 945.5440, 783.4904, 621.4373, 459.3847 | notoginsenoside-FZ or isomer |
| **56** | 17.58 | 955.4923[a] | $C_{48}H_{76}O_{19}$ | 1.54 | 200.20 | –H | 955.4915, 793.4887, 613.3763, 569.3852, 523.3783, 497.3650, 455.3536 | ginsenoside Ro |
| **59** | 18.13 | 1193.5970[a] | $C_{57}H_{94}O_{26}$ | 0.49 | 265.46 | –H | 1107.5965, 945.5437, 783.4904, 621.4378, 459.3853 | malonylginsenoside Rb1 |
| **61** | 18.40 | 1255.6320[a] | $C_{58}H_{98}O_{26}$ | 0.80 | 368.97 | +HCOO | 1209.6268, 1077.5853, 945.5437, 783.4903, 765.4802, 621.4368, 459.3845 | notoginsenoside Fc |
| **64** | 19.34 | 1123.5890[a] | $C_{53}H_{90}O_{22}$ | 0.37 | 275.15 | +HCOO | 1077.5830, 945.5417, 783.4889, 653.4628, 537.3445, 459.3856 | ginsenoside Rc |
| **66** | 19.80 | 1255.6320[a] | $C_{58}H_{98}O_{26}$ | 0.32 | 363.00 | +HCOO | 1209.6260, 1077.5844, 945.5436, 783.4892, 621.4370, 459.3845 | ginsenoside Ra1 |
| **68** | 20.14 | 1193.5950[b] | $C_{57}H_{94}O_{26}$ | –0.68 | – | –H | 1149.6070, 1107.5955, 945.5430, 783.4901, 621.4373, 459.3847 | malonylfloralginsenoside Rb2 or isomer |
| **71** | 20.92 | 1387.6720[a] | $C_{63}H_{106}O_{30}$ | –1.50 | 381.91 | +HCOO | 1341.6676, 1209.6257, 1077.5843, 945.5428, 783.4897, 621.4367, 459.3850 | notoginsenoside S |
| **73** | 21.19 | 683.4455[a] | $C_{36}H_{62}O_{9}$ | –0.48 | 275.30 | +HCOO | 637.4327, 621.4337, 475.3801, 391.2873 | ginsenoside F1 |
| **75** | 21.62 | 1295.6270[b] | $C_{61}H_{100}O_{29}$ | –0.42 | 374.67 | –H | 1209.6284, 1077.5854, 945.5435, 783.4897, 621.4372, 459.3849 | PPD-mal-2xyl-3glc |

| | | | | | | | | |
|---|---|---|---|---|---|---|---|---|
| **77** | 21.97 | 1163.5860[b] | $C_{56}H_{92}O_{25}$ | 0.44 | – | –H | 1077.5853, 945.5437, 783.4878, 621.4337, 459.3830 | malonylginsenoside Rb2 or isomer |
| **84** | 22.28 | 1123.5890[a] | $C_{53}H_{90}O_{22}$ | 0.04 | 359.67 | +HCOO | 1077.5821, 945.5406, 783.4878, 621.4378, 459.3845 | ginsenoside Rb2 |
| **85** | 22.58 | 1295.6250[b] | $C_{61}H_{100}O_{29}$ | –1.89 | 362.70 | –H | 1209.6273, 1077.5848, 945.5426, 783.4900, 621.4372, 459.3836 | PPD-mal-2xyl-3glc |
| **87** | 23.13 | 1163.5850[b] | $C_{56}H_{92}O_{25}$ | –0.77 | – | –H | 1077.5843, 945.5442, 783.4853, 621.4353, 459.3890 | malonylfloralginsenoside Rc3 or isomer |
| **90** | 23.43 | 1123.5890[a] | $C_{53}H_{90}O_{22}$ | –1.29 | 347.37 | +HCOO | 1077.5834, 945.5422, 783.4891, 621.4364, 459.3842 | ginsenoside Rb3 |
| **94** | 24.24 | 1295.6270[b] | $C_{61}H_{100}O_{29}$ | –0.66 | – | –H | 1209.6274, 1077.5854, 945.5438, 783.4898, 621.4371, 459.3848 | PPD-mal-2xyl-3glc |
| **97** | 24.54 | 1123.6080[b] | $C_{53}H_{90}O_{22}$ | 1.30 | 357.12 | +HCOO | 1077.5865, 945.5421, 783.4893, 621.4356, 459.3807 | notoginsenoside L or isomer |
| **98** | 24.60 | 1255.6310[b] | $C_{58}H_{98}O_{26}$ | –1.45 | 284.63 | +HCOO | 1209.6256, 1077.5844, 945.5424, 783.4896, 621.4362, 459.3839 | notoginsenoside-FZ or isomer |
| **104** | 26.10 | 1163.5840[b] | $C_{56}H_{92}O_{25}$ | –1.11 | 279.06 | –H | 1077.5843, 945.5430, 783.4901, 621.4371, 459.3841 | malonylfloralginsenoside Rc2 or isomer |
| **108** | 27.10 | 1163.5840[b] | $C_{56}H_{92}O_{25}$ | –1.50 | 267.37 | –H | 1077.5833, 945.5418, 783.4889, 621.4364, 459.3841 | malonylfloralginsenoside Rc1 or isomer |
| **113** | 27.80 | 1163.5850[b] | $C_{56}H_{92}O_{25}$ | –0.16 | 278.78 | –H | 1077.5831, 945.5420, 783.4892, 621.4264, 459.3834 | malonylfloralginsenoside Rc4 or isomer |
| **127** | 30.27 | 1031.5440[a] | $C_{51}H_{84}O_{21}$ | 1.00 | 340.41 | –H | 945.5435, 783.4904, 621.4387, 459.3845 | malonylginsenoside Rd |
| **140** | 32.36 | 1033.5570[b] | $C_{50}H_{84}O_{19}$ | 1.01 | 213.18 | +HCOO | 987.5544, 945.5428, 783.4905, 621.4370, 459.3838 | quinquenoside III or isomer |
| **179** | 34.58 | 961.5373[a] | $C_{47}H_{80}O_{17}$ | –2.92 | 322.31 | +HCOO | 915.5296, 783.4889, 621.4361, 459.3817 | notoginsenoside Fe |
| **185** | 35.36 | 961.5381[a] | $C_{47}H_{80}O_{17}$ | 0.32 | 322.73 | +HCOO | 915.5321, 621.4382, 459.3866 | ginsenoside Rd2 |
| **186** | 35.64 | 1001.5330[b] | $C_{50}H_{82}O_{20}$ | 0.63 | 319.34 | –H | 915.5328, 783.4955, 621.4380, 459.3850 | PPD-mal-xyl-2glc |
| **189** | 35.45 | 1093.5800[b] | $C_{53}H_{90}O_{23}$ | 0.12 | 338.01 | +HCOO | 1047.5758, 915.5321, 783.4890, 621.4381, 459.3849 | notoginsenoside P or isomer |
| **196** | 36.25 | 1001.5330[b] | $C_{50}H_{82}O_{20}$ | –0.07 | 331.19 | –H | 915.5347, 783.4903, 621.4380, 459.3852 | PPD-mal-xyl-2glc |
| **202** | 36.76 | 829.4961[a] | $C_{42}H_{72}O_{13}$ | 0.73 | 306.82 | +HCOO | 783.4929, 621.4385, 459.3844, 375.2900 | ginsenoside F2 |
| **217** | 39.39 | 799.4856[b] | $C_{42}H_{72}O_{14}$ | 0.88 | 304.95 | +HCOO | 753.4789, 621.4361, 459.3843 | gypenoside XIII or isomer |

Note: The number is the same as that in the identification list Tab.1 of the flower buds of *Panax notoginseng*; a: Identification assisted with reference compounds comparison; b: Tentative characterization by analyzing the high-accuracy $MS^2$ data.

**Table S4.** Information for 11 metabolites identified from rat plasma.

| No. | Observed RT (min) | Observed *m/z* | Formula | Mass error (ppm) | CCA (Å[2]) | Adducts | ESI-MS[2] | Metabolic pathways | Identification |
|---|---|---|---|---|---|---|---|---|---|
| **M1** | 5.25 | 977.5306[b] | $C_{48}H_{82}O_{20}$ | 1.07 | 324.23 | –H | 931.5275, 827.5607, 799.4864, 637.4317, 475.3791 | ginsenoside Re+2O | PPT+2O+2glc+rha |
| **M2** | 15.97 | 1107.5940[a] | $C_{54}H_{92}O_{23}$ | 2.88 | 350.46 | –H | 945.5427, 783.4901, 621.4381, 459.3851 | notoginsenoside Fa-xyl/notoginsenoside R4-xyl | ginsenoside Rb1 |
| **M3** | 18.45 | 1209.6230[b] | $C_{58}H_{98}O_{26}$ | –3.10 | 369.83 | –H | 1077.5825, 945.5409, 783.4882, 621.4354, 459.3841 | notoginsenoside Fh1-xyl | ginsenoside Ra1/notoginsenoside-FZ isomer |
| **M4** | 19.35 | 1077.5820[b] | $C_{53}H_{90}O_{22}$ | –1.90 | 342.67 | –H | 945.5422, 783.4883, 621.4358, 459.3844 | notoginsenoside Fc-xyl/notoginsenoside Fa-glc | vinaginsenoside R7 |
| **M5** | 23.41 | 783.4890[b] | $C_{42}H_{72}O_{13}$ | 3.90 | 308.67 | –H | 783.4902, 708.3636, 642.3125 | ginsenoside Re-glc | ginsenoside Rg2 |
| **M6** | 26.10 | 793.4401[b] | $C_{44}H_{66}O_{14}$ | 4.82 | 287.58 | –H | – | ginsenoside Ro-glc | zingibroside R1 |
| **M7** | 28.38 | 991.5469[b] | $C_{48}H_{82}O_{18}$ | –0.80 | 327.01 | +HCOO | 945.5423, 783.4890, 621.4370, 459.3858 | ginsenoside Rb2-ara | ginsenoside Rd |
| **M8** | 28.50 | 991.5469[b] | $C_{48}H_{82}O_{18}$ | –2.00 | 325.96 | +HCOO | 945.5423, 783.4890, 621.4370, 459.3858 | ginsenoside Rb1-glc | gypenoside XVII |
| **M9** | 34.58 | 915.5311[b] | $C_{47}H_{80}O_{17}$ | 1.41 | 319.12 | –H | 837.4027, 716.3645, 621.4389, 459.3852 | notoginsenoside R4-2glc | PPD+mal+2glc+Xyl |
| **M10** | 37.55 | 869.4884[b] | $C_{45}H_{74}O_{16}$ | 3.47 | 308.83 | –H | 845.6852 | malonylginsenoside Rd-glc | PPD+mal+2glc |
| **M11** | 37.88 | 885.5227[b] | $C_{46}H_{78}O_{16}$ | 3.02 | 321.52 | –H | 830.3231, 753.4815 | notoginsenoside P-glc | PPD+glc+2xyl |

a: Identification assisted with reference compounds comparison; b: Tentative characterization by analyzing the high-accuracy MS[2] data.

**Table S5.** CCS prediction of isomers in rat plasma based on ALLCCS and CCSbase.

| No. | Observed RT (min) | Observed m/z | Formula | Adducts | Observed CCA (Å²) | Predicted CCA (Å²) ALLCCS[M–H]⁻ | ALLCCS [M–H+HCOOH]⁻ | CCSbase [M–H]⁻ | Identification |
|---|---|---|---|---|---|---|---|---|---|
| **31** | **10.81** | **1285.6450[a]** | **$C_{59}H_{100}O_{27}$** | **+HCOO** | **372.56** | – | – | 331.9 | notoginsenosides R4 |
| 38 | 12.84 | 1285.6410[a] | $C_{59}H_{100}O_{27}$ | +HCOO | 372.92 | – | – | 331.9 | notoginsenoside Fa |
| 48 | 15.40 | 1255.6320[a] | $C_{58}H_{98}O_{26}$ | +HCOO | 365.25 | – | – | 325.4 | notoginsenoside FP2 |
| 61 | 18.4 | 1255.6320[a] | $C_{58}H_{98}O_{26}$ | +HCOO | 368.97 | – | – | 327.0 | notoginsenoside Fc |
| 66 | 19.8 | 1255.6320[a] | $C_{58}H_{98}O_{26}$ | +HCOO | 363.00 | – | – | 327.0 | ginsenoside Ra1 |
| 84 | 22.28 | 1123.5890[a] | $C_{53}H_{90}O_{22}$ | +HCOO | 359.67 | 273.9 | 287.9 | 301.4 | ginsenoside Rb2 |
| 90 | 23.43 | 1123.5890[a] | $C_{53}H_{90}O_{22}$ | +HCOO | 347.37 | 273.9 | 287.9 | 301.4 | ginsenoside Rb3 |
| 97 | 24.54 | 1123.6080[b] | $C_{53}H_{90}O_{22}$ | +HCOO | 357.12 | 273.9 | 287.9 | 301.4 | notoginsenoside L |
| M4 | 19.35 | 1077.5820[b] | $C_{53}H_{90}O_{22}$ | –H | 342.67 | 271.7 | 285.7 | 301.4 | vinaginsenoside R7 |
| 179 | 34.58 | 961.5373[a] | $C_{47}H_{80}O_{17}$ | +HCOO | 322.31 | 252.0 | 266.1 | 271.0 | notoginsenoside Fe |
| 185 | 35.36 | 961.5381[a] | $C_{47}H_{80}O_{17}$ | +HCOO | 322.73 | 252.1 | 266.2 | 271.9 | ginsenoside Rd2 |
| M7 | 28.38 | 991.5469[b] | $C_{48}H_{82}O_{18}$ | +HCOO | 327.01 | 255.4 | 269.6 | 274.4 | ginsenoside Rd |
| M8 | 28.50 | 991.5469[b] | $C_{48}H_{82}O_{18}$ | +HCOO | 325.96 | 257.6 | 269.6 | 274.4 | gypenoside XVII |
| 6 | 5.65 | 991.5484[a] | $C_{48}H_{82}O_{18}$ | +HCOO | 327.00 | 256.4 | 270.5 | 274.5 | ginsenoside Re |
| 202 | 36.76 | 829.4961[a] | $C_{42}H_{72}O_{13}$ | +HCOO | 306.82 | 237.5 | 250.3 | 252.6 | ginsenoside F2 |
| M5 | 23.41 | 783.4890[b] | $C_{42}H_{72}O_{13}$ | –H | 308.67 | 239.0 | 251.8 | 251.6 | ginsenoside Rg2 |

Note: The number is the same as that in the identification list Tab.1 and Tab.3.; a: Identification assisted with reference compounds comparison; b: Tentative characterization by analyzing the high-accuracy MS² data.
